# Supplementary material for: An epigenetic biomarker of aging for lifespan and healthspan
Source: Aging (Albany NY). 2018 Apr 17;10(4):573–91. doi: 10.18632/aging.101414 (PMC5940111; doi:10.18632/aging.101414)
Supplement: Supplement 1 [file aging-10-101414-s001.pdf]

Additional file for

" An epigenetic biomarker of aging for lifespan and healthspan"

by Morgan E. Levine et al.

SUPPLEMENTARY METHODS

Overview of the phenotypic age estimate

Using the NHANES training data, we applied a Cox penalized regression model—where the hazard of aging-related mortality (mortality from diseases of the heart, malignant neoplasms, chronic lower respiratory disease, cerebrovascular disease, Alzheimer's disease, Diabetes mellitus, nephritis, nephrotic syndrome, and nephrosis) was regressed on forty-two clinical markers and chronological age to select variables for inclusion in our phenotypic age score. Ten-fold cross-validation was employed to select the parameter value, lambda, for the penalized regression. In order to develop a sparse phenotypic age estimator (the fewest biomarker variables needed to produce robust results) we selected a lambda of 0.0192, which represented a one standard deviation increase over the lambda with minimum mean-squared error during cross-validation (**Supplementary Fig. S10**). Of the forty-two biomarkers included in the penalized Cox regression model, this resulted in ten variables (including chronological age) that were selected for the phenotypic age predictor.

These nine biomarkers and chronological age were then included in a parametric proportional hazards model based on the Gompertz distribution. Based on this model, we estimated the 10-year (120 months) mortality risk of the j-the individual based on the cumulative distribution function

$$MortalityScore_j = CDF(120, x_j) = 1 - e^{-e^{x_j b}(\exp(120*\gamma)-1)/\gamma}$$

where xb= represents the linear combination of biomarkers from the fitted model (**Supplementary Table S1**)

Next, the mortality score was converted into units of years using the following equation

$$PhenotypicAge_j = 141.50225 + \frac{\ln(-.00553 * \ln(1 - MortalityScore_j)))}{0.090165}$$

Statistical details on the Gompertz proportional hazards model for phenotypic age estimation

The Gompertz regression is parameterized only as a proportional hazards model. This model has been extensively used extensively for modeling mortality data. The Gompertz distribution implemented is the two-parameter function as described in Lee and Wang (2003)[1], with the following hazard and survivor functions:

$$h(t) = \lambda \exp(\gamma t)$$

$$S(t) = \exp\{-\lambda\gamma^{-1}(e^{\gamma t} - 1)\}$$

The covariates of the j-th individual are including in the model using the following parametrization: $\lambda_j = \exp(\mathbf{x}_j \boldsymbol{\beta})$ which implies that the baseline hazard is given by $h_0(t) = \exp(\gamma t)$ where γ is an ancillary parameter to be estimated from the data. The cumulative distribution function of the Gompertz model is given by

CDF(t,x)=1-exp(-exp(xb) (exp(γt)-1)/γ)

where t denotes time (here in units of months) and $\mathrm{xb} = \sum_{u=1}^{p} x^u b^u + b^0$.

The Gompertz regression analysis resulted in coefficient values and parameter values (Table 1, Table S1) and γ =0.0076927.

In step 2, we used the cumulative distribution function of the Gompertz model to estimate the 120-month mortality risk of each individual. Thus, CDF(t=120,x_j) denotes the probability that the j-th individual will die within the next 120 months.

In step 3, carried out another parametric proportional hazards model analysis with Gompertz distribution, but only including chronological age as a IV. We will refer to this analysis as the univariate Gompertz regression model since it only involved one covariate (age). The resulting estimate of the cumulative distribution function CDF.univariate(t,age)

$$\mathrm{CDF.univariate}(t, \mathrm{age}) = 1 - e^{\left\{-e^{(\mathrm{age}*b^1+b^0)}\gamma^{-1}(e^{\gamma t}-1)\right\}}$$

allowed us to estimate the probability that the j-th individual with die within 120 months as follows CDF.univariate(120,age_j) where age_j is the age of the j-th individual.

In step 4, we solved the equation CDF(120,x_j)=CDF.univariate(120,age_j) for the variable age_j. The resulting solution for the j-th individual, referred to as PhenotypicAge, is given by

$$\mathrm{PhenotypicAge}_j = 141.50225 + \frac{\ln(-0.00553 * \ln(1 - \mathrm{CDF}(120, x_j)))}{0.090165}$$

Data used to generate DNAm PhenoAge

Participants ages 20 and over in NHANES III (1988-94) were used as the training sample to develop a new and improved measure of phenotypic aging (n=9,926), while participants ages 20 and over in NHANES IV (1999-2014) were used to validate the association between phenotypic aging and age-related morbidity and mortality (n=6,209). Overall, NHANES III had available mortality follow-up for up to 23 (n= deaths) and NHANES IV had available mortality follow-up for up to 17 years (n= deaths). InCHIANTI included longitudinal (two time-points—1998 and 2007) phenotypic and DNAm data on n=456 male and female participants, ages 21-91 in 1998, and 30-100 in 2007. Participants from WHI included 2,107 post-menopausal women, who were ages 50-80 at baseline and were followed-up for just over 20 years.

DNA methylation data

All but one cohort used the DNAm data sets used the Illumina Infinium 450K platform. However, the data from the Jackson Heart Study were generated on the EPIC array.

The Illumina BeadChips measures bisulfite-conversion-based, single-CpG resolution DNAm levels at different CpG sites in the human genome. These data were generated by following the standard protocol of Illumina methylation assays, which quantifies methylation levels by the β value using the ratio of intensities between methylated and un-methylated alleles. Specifically, the β value is calculated from the intensity of the methylated (M corresponding to signal A) and un-methylated (U corresponding to signal B) alleles, as the ratio of fluorescent signals $\beta = Max(M,0)/[Max(M,0)+Max(U,0)+100]$. Thus, β values range from 0 (completely un-methylated) to 1 (completely methylated). For WHI we used background corrected beta values, while InCHIANTI and the JHS data were normalized using the NOOB method [2].

Main Validation Studies

A number of validation studies were used to test the associations between DNAm PhenoAge and various aging-related traits. WHI (samples 1 and 2), FHS, and NAS comprised the main validation data for mortality and morbidity analyses. The two separate WHI subsamples were aggregated for our study within the WHI (BA23 and AS315). WHI sample 1 included 2,091 for whom complete data was available for DNAm PhenoAge, morbidity, mortality, and confounder variables. Participants were part of a subsample from the Women's Health Initiative (WHI), who were enrolled as part of an integrative genomics study, with the primary focus on identifying determinants of CHD risk as detailed in [3]. This sample included women ages 50-79 at baseline, with an overrepresentation of racial/ethnic minorities. About half of the samples developed coronary heart disease after the baseline measurement. The integrative genomics subsample employed a case-control sampling design. All incident cases and controls were required to have already undergone genome wide genotyping at baseline as well as profiling of seven cardiovascular biomarkers, as dictated by the aims of other ancillary WHI studies.

The second WHI data set was part of the Women's Health Initiative – Epigenetic Mechanisms of PM-Mediated CVD (WHI-EMPC, AS315) is an ancillary study of epigenetic mechanisms underlying associations between ambient particulate matter (PM) air pollution and cardiovascular disease (CVD) in the Women's Health Initiative clinical trials (CT) cohort. The WHI-EMPC study population is a stratified, random sample of 2,200 WHI CT participants who were examined between 1993 and 2001; had available buffy coat, core analytes, electrocardiograms, and ambient concentrations of PM; but were not taking anti-arrhythmic medications at the time. As such, WHI-EMPC is representative of the larger, multiethnic WHI CT population from which it was sampled: n=68,132 participants aged 50-79 years who were randomized to hormone therapy, calcium/vitamin D supplementation, and / or dietary modification in 40 U.S. clinical centers at the baseline exam (1993-1998) and re-examined in the fasting state one, three, six, and nine years later [4].

The Normative Aging Study (NAS)

The US Department of Veterans Affairs (VA) Normative Aging Study (NAS) is an ongoing longitudinal cohort established in 1963, which included men who were 21-80 years of age and free of known chronic medical conditions at entry[5]. Participants were subsequently invited to medical examinations every three to five years. At each visit, participants provided information on medical history, lifestyle, and demographic factors, and underwent a physical examination and laboratory tests. DNA samples were collected from between 1999-2007 from the 675 active participants and used for DNAm analysis. We excluded 18 participants who were non-whites or had missing information on race, leaving a total of 657 individuals.

Official death certificates were obtained for decedents from the appropriate state health department and were reviewed by a physician. Experienced research nurse coded the cause of death using ICD-9. Both participant deaths and cause of death are routinely updated by the research team and last update available was December 31, 2013.

Jackson Heart Study

The JHS is a large, population-based observational study evaluating the etiology of cardiovascular, renal, and respiratory diseases among African Americans residing in the three counties (Hinds, Madison, and Rankin) that make up the Jackson, Mississippi metropolitan area [6]. Data and biologic materials have been collected from 5306 participants, including a nested family cohort of 1,498 members of 264 families. The age at enrollment for the unrelated cohort was 35-84 years; the family cohort included related individuals >21 years old. Participants provided extensive medical and social history, had an array of physical and biochemical measurements and diagnostic procedures, and provided genomic DNA during a baseline examination (2000-2004) and two follow-up examinations (2005-2008 and 2009-2012). The study population is characterized by a high prevalence of diabetes, hypertension, obesity, and related disorders. Annual follow-up interviews and cohort surveillance are ongoing.

In our analysis, we used Illumina EPIC array data from n=1756 African Americans (n=1203 women and n=653 men) that were generated as part of project JHS ancillary study ASN0104. The blood samples were collected at the baseline of the study (visit 1). At the time of the blood draw, the individuals ranged from 22 to 93 (median age 57). At the time of the last follow up, 282 individuals were known to be deceased. The median number of years of follow up (time to death or last follow up) was 12.2 years (ranging from 0.14 to 14.5 years).

DNAmPhenoAgeAccel ranged from -19 to 38. 4.7 and 4.9 percent of individuals had a value of DNAmPhenoAgeAccel larger than 10 or smaller than -10, respectively.

Framingham Heart Study Offspring Cohort (FHS)

The Framingham Heart Study (FHS) Offspring Cohort began enrollment in 1971 and included 5,124 offspring and spouses of the offspring of the FHS original cohort. Participants were eligible for the current study if they attended the eighth examination cycle (2005-2008) and consented to having their DNA to be used for genetic research. All participants provided written informed consent at the time of each examination visit. The study protocol was approved by the Institutional Review Board at Boston University Medical Center (Boston, MA). The FHS data are available in dbGaP (accession number "phs000724.v2.p9").

Deaths among FHS participants that occurred prior to January 1, 2013 were ascertained using multiple strategies, including routine contact with participants for health history updates, surveillance at the local hospital and in obituaries of the local newspaper, and queries to the National Death Index. Death certificates, hospital and nursing home records prior to death, and autopsy reports were requested. When cause of death was undeterminable, the next of kin were interviewed. The date and cause of death were reviewed by an endpoint panel of 3 investigators.

Early menopause and blood methylation data

We previously reported an association between early menopause and epigenetic age acceleration (based on Horvath DNAm Age) [7]. We used the same data from BA23 of the Women's Health Initiative (described above) to replicate this finding using DNAm PhenoAge acceleration.

Alzheimer's disease and brain methylation data from the Religious Order Study

A number of other samples were used to validate the accuracy of DNAm PhenoAge in various tissues or for case-control studies. For instance, the Religious Order Study (ROS) and the Memory and Aging Project (MAP) were used to test the association between DNAm PhenoAge in DLPCTX and Alzheimer’s disease

and/or neuropathology. Both are longitudinal community based cohort studies of aging and dementia. The majority of participants in both studies are 75-80 years old at baseline with no known dementia. Inclusion in the studies requires participants to consent to undergoing annual clinical evaluations as well as postmortem organ donation. The ROS sample includes Catholic priests, nuns, and brothers from across the United States, whereas the MAP sample includes a more general community based population from northeastern Illinois.

Breast cancer risk and blood methylation data from the EPIC study

We previously reported an association between intrinsic epigenetic age acceleration (based on the Horvath DNAm age estimator) and breast cancer risk [8]. Using the same data, we studied whether DNAmPhenoAgeAcceleration in blood also predicts incident breast cancer. The Illumina 450K DNA methylation data came from a nested case-control study embedded in the European Prospective Investigation into Cancer and Nutrition (EPIC) cohort (n = 960 females) [8]. The 960 females from the EPIC cohort included 480 incident breast cancer cases. According to Ambatipudi (2017), the main criteria for selection of case/control pairs included: (1) a balanced representation of the main subtypes of breast cancer, and (2) representation of recruiting centres. One control participant was randomly assigned for each patient with breast cancer from appropriate risk sets consisting of all cohort participants alive and free of cancer (except for non-melanoma skin cancer) at the time of diagnosis (and hence, age) of the index case. Matching criteria were: centre, length of follow-up, age at blood collection, time of blood collection, fasting status, menopausal status, menstrual cycle day and current use of contraceptive pill/hormone replacement therapy.

Offspring of Italian semi-supercentenarians

Semi-supercentenarians (aged between 105-109 years) are of great interest to aging researchers because they often managed to avoid the onset of major age-related diseases. We previously reported that the offspring of semi-supercentenarians are epigenetically younger than age matched controls (i.e. individuals of non-centenarians) using the Horvath DNAm age estimator [9]. Using the same blood methylation data, we revisited this analysis using DNAmPhenoAgeAcceleration.

Claudio Franceschi and Paolo Garagnani generated Illumina 450K DNA methylation levels from peripheral blood mononuclear cells (PBMCs) from Italian families comprised of 82 semi-supercentenarians (mean age: 105.6 ± 1.6 years), 63 semi-supercentenarians' offspring (mean age: 71.8 ± 7.8 years), and 47 age-matched controls (mean age: 69.8 ± 7.2 years). We find a marginally significant reduction of approximately 2.4 years for the DNAm PhenoAge of semi-super centenarian offspring, relative to controls (β=-2.40, p=0.065) (comprised of 63 semi-supercentenarians' offspring and 47 age matched controls). No significant difference could be observed for estimated blood cell counts (such as naïve or exhausted cytotoxic T cells or helper T cells).

Dementia status versus blood methylation

We evaluated whether DNAm PhenoAge in blood relates to clinically diagnosed dementia in living individuals using a blood data set from [10]. Results suggest that those with presumed Alzheimer's disease (AD, n=154) and/or frontotemporal dementia (FTD, n=116) have significantly higher DNAm PhenoAge compared to non-demented (n=334) individuals (P=2.2E-2). Patients were enrolled as part of a large genetic study in neurodegenerative dementia (Genetic Investigation in Frontotemporal Dementia, GIFT) at the UCSF Memory and Aging Center (UCSF-MAC) [10]. The blood DNA methylation data were generated on the Illumina 450K array.

Down syndrome versus blood methylation

We previously reported that individuals with Down syndrome are epigenetically older than age matched controls according to the Horvath DNAm age estimator [11]. Using the same blood data sets, we replicated the results for DNAm PhenoAge. Leukocyte data set 1 (measured on the Illumina 27K platform) involved 35 participants with DS and 21 controls (mean age 43 ranging from 22 to 64) that were ascertained through the New York State developmental disability service system as well as agencies in New Jersey, Connecticut and Northern Pennsylvania [12]. Blood data set 2 (measured on the Illumina 450K array) involved 29 individuals with Down syndrome, their mothers (DSM) and their unaffected siblings (DSS) [11]. The individuals investigated in this study were recruited in Emilia-Romagna region (Bologna and Ferrara provinces), Italy. In our original article, we showed that DS individuals also exhibited epigenetic age acceleration in brain tissue according to the Horvath DNAm age estimator [11]. By contrast, DNAm PhenoAge did not reveal a significant epigenetic age acceleration effect in the small brain data set (n=15 brain samples from DS and 54 controls).

HIV infection and blood methylation

We previously reported that HIV infection is associated with accelerated epigenetic age acceleration according to the Horvath DNAm age estimator. Here we used the same blood data sets to revisit this analysis with DNAm PhenoAge. Specifically, we used two publicly available blood DNA methylation data from HIV+ individuals and HIV- controls from [13]. All data sets were generated with the Illumina 450K array. The first dat set involved Peripheral blood mononuclear cells (PBMCs) isolated from 92 individuals were evaluated. The 24 HIV+cases had a mean age of 49 years (range, 29–67 years). The 68 controls had a mean age of 36 years (range, 18–74 years). The second data also involved PBMCs from HIV+ cases and HIV negative controls. The 23 cases had a mean age of 45 years (range, 24–68 years). The 69 controls had a mean age of 51 years (range, 35–64 years). The HIV+ cases were ascertained by the National Neurological AIDS Bank study or Multicenter AIDS Cohort Study in Los Angeles.

Parkinson's disease and blood methylation

We previously reported that Parkinson's disease is associated with a weak age acceleration effect according to both the Horvath and Hannum DNAm age estimators [14]. Using the same data, we revisited this analysis with DNAm PhenoAge. We observe a suggestive relationship between DNAm PhenoAge in blood and Parkinson's disease status (p=0.028) the n=508 individuals (n=289 PD cases and n=219 controls) of European ancestry. The blood DNA methylation data were measured on the Illumina 450K array. The blood samples came from the Parkinson's disease, Environment, and Genes (PEG) case-control study [15]. The PEG study is a large population-based study of Parkinson's disease of mostly rural and township residents of California's central valley. Cases were identified with the help of local neurologists, clinics, and community outreach and controls were randomly sampled from Medicare lists and residential tax assessor's records.

Estimation of blood cell counts based on DNAm levels

We estimate blood cell counts using two different software tools. First, Houseman's estimation method [16] was used to estimate the proportions of CD8+ T cells, CD4+ T, natural killer, B cells, and granulocytes (also known as polymorphonuclear leukocytes). Second, the Horvath method, implemented in the advanced analysis option of the epigenetic clock software [13, 17], was used to estimate the percentage of exhausted CD8+ T cells (defined as CD28-CD45RA-), the number (count) of naïve CD8+ T cells (defined as CD45RA+CCR7+) and plasmablasts. We and others have shown that the estimated blood cell counts have moderately high correlations with corresponding flow cytometric measures [16, 18].

Heritability Analysis using SOLAR

The polygenic model implemented in SOLAR (Sequential Oligogenic Linkage Analysis Routine) software [19] was used to estimate heritability of DNAm PhenoAge in the FHS pedigree cohort based on the known KINSHIP coefficients. Heritability is defined as the total proportion of phenotypic variance attributable to genetic variation in the polygenic model. The polygenic model was adjusted gender and chronological age. GCTA -We performed the REML analysis [20, 21] to estimate the heritability of PhenoAgeAccel, using the postmenopausal women of the WHI cohort to estimate heritability of age acceleration. The WHI sub-studies were genotyped on different platforms. In order to combine the genotype data across the studies from WHI EMPC and WHI BA23, we converted the MaCH dosage format into PLINK format with best guess genotypes and used both genotyped and imputed markers for analysis. We only used the overlapped markers existing in all studies (such that SNP missing rate=0) and controlled the quality of SNPs based on MAF > 0.05, Hardy Weinberg equilibrium (HWE) P > 0.0001, and MaCH r^2 > 0.8, yielding approximately 4M markers available for analysis. All analyses were adjusted for 4 principal components (PC).

Enrichment Analysis using GREAT

We applied the GREAT analysis to analyze the functional involvements of cis-regulatory regions of the 513 CpG sites. Of the 513 CpG sites, 242 are positively correlated with chronological age in blood and 271 are negatively correlated with chronological age in blood. We used three different sets of genes as input—all, those co-locating to CpGs with positive age associations, and those co-locating to CpGs with negative age associations. Enrichment was assessed using the whole genome as background. The GREAT software performs both binomial test over genomic regions and hypergeometric test over genes when using a whole genome background.

Estimation of neuronal proportions in brain tissues

The CETS R package[22] was used to estimate the proportion of neurons based on DNA methylation data. We independently confirmed the high accuracy of the CETS algorithm by applying it sorted neurons, which led to estimates of the proportion of neurons in excess of 0.99.

Gene expression analysis of AgeAccelPheno

DNA methylation and gene expression from the Monocyte[23] and FHS datasets were adjusted for array effects using ComBat from the sva R package. The AAP variable was adjusted using linear modeling for site, race (in the Monocyte dataset), family structure (in the FHS dataset), gender, and cell proportion estimates where designated (Houseman estimates excluding the major cell type to avoid multi-colinearity: CD8T, CD4T, NK, Bcell, Mono (excluded in the Monocyte dataset), Gran (excluded in the FHS dataset). The individual gene expression probe levels where tested for associations with AAP using a robust correlation measure (biweight midcorrelation). The top 5% of genes positively and negatively correlated with the AAP where analyzed for GO term enrichment using the topGO R package.

The purified monocyte samples were from the April 2010-February 2012 examination of 1,264 randomly selected MESA participants (55-94 years old, Caucasian (47%), African American (21%) and Hispanic (32%), female 51%) from four MESA field centers (Baltimore, MD; Forsyth County, NC; New York, NY; and St. Paul, MN). The study protocol was approved by the Institutional Review Boards at Johns Hopkins Medical Institutions, University of Minnesota, Columbia University Medical Center, and Wake Forest University Health Sciences. All participants signed informed consent. In depth details about the purification process can be found in the original publication (doi:10.1038/ncomms6366). Briefly, mononuclear cells were isolated from peripheral blood that was collected in sodium heparin tubes. Monocytes (>90% purity by FACS) were isolated using anti-CD14 monoclonal antibody coated beads. The methylome and transcriptome of the monocyte samples were profiled using the Illumina

HumanMethylation450 BeadChip and Illumina HumanHT-12 v4 Expression BeadChip platforms with standard Illumina protocols.

SUPPLEMENTARY RESULTS

DNAm PhenAge and other aging-related morbidity outcomes

Additional independent replication data was used to test for associations with other aging outcomes, which have previously been shown to relate to the first generation of epigenetic biomarkers [8-11, 13, 14]. Using the five studies described above, we find that women with higher DNAm PhenoAge tended to have an earlier age at menopause (Meta P-value=1.32E-2). Among the 527 women who were cancer free at age 50, accelerated DNAm PhenoAge in blood predicts incident breast cancer (OR: 1.037, p=0.033) using data from [8]. We find a marginally significant reduction of approximately 2.4 years for the DNAm PhenoAge of semi-super centenarian offspring, relative to controls (β=-2.40, p=0.065) in a relatively small blood data set (comprised of 63 semi-supercentenarians' offspring and 47 age matched controls from [9]). We evaluated whether DNAm PhenoAge relates to clinically diagnosed dementia in living individuals using a blood data set from [10]. Results suggest that those with presumed Alzheimer's disease (AD, n=154) and/or frontotemporal dementia (FTD, n=116) have significantly higher DNAm PhenoAge compared to non-demented (n=334) individuals (P=2.2E-2), and the strength of the association is further increased (P=9.4E-3) when limiting the sample to those ages 75 and older. We also find that DNAm PhenoAge relates to Down syndrome in two separate blood methylation datasets (p=0.0046, n=56; and p=4.0E-11, n=87) described in [11]. We find that HIV infection is associated (p=6E-6 and p=8.6E-6) with accelerated DNAm PhenoAge in two blood datasets (n=92 and n=92) described in [13]. Finally, we observe a suggestive relationship between DNAm PhenoAge in blood and Parkinson's disease status (p=0.028) in a large data set (n=508) of individuals of European ancestry from [14].

DNAm PhenAge and social, behavioral, and demographic factors

In evaluating the relationship between DNAm PhenoAge and additional behavioral, social, and demographic characteristics we observe significant differences between racial/ethnic groups (p=5.1E-5), with non-Hispanic blacks having the highest DNAm PhenoAge on average, and non-Hispanic whites having the lowest (**Supplementary Fig. S5)**. We also find evidence of social gradients in DNAm PhenoAge, such that those with higher education (p=6E-9) and higher income (p=9E-5) appear younger. DNAm PhenoAge relates to exercise and dietary habits, such that increased exercise (p=7E-5) and markers of fruit/vegetable consumption (such as carotenoids, p=2E-27) are associated with lower DNAm PhenoAge (**Supplementary Fig. S6A & Supplementary Fig. S6B)**. Cross sectional studies in the WHI also revealed that DNAmPhenoAge acceleration is positively correlated with C-reactive protein (r=0.18, p=5E-22, **Supplementary Fig. S6B**), insulin (r=0.15, p=2E-20), glucose (r=0.10, p=2E-10), triglycerides (r=0.09, p=5E-9), waist to hip ratio (r=0.15, p=5E-22) but it is negatively correlated with the "good" cholesterol HDL (r=-0.09, p=7E-9).

Effect of obesity on liver and adipose tissue

Using the Horvath DNAm age measure, we previously found that body mass index correlated with epigenetic age acceleration in two independent human liver samples (r=0.42 and r=0.42 in liver data sets 1 and 2, respectively) [24]. Using the same data, we replicated this finding using the new measure of PhenoAge acceleration (r=0.32, p=0.011 and r= 0.48 p=7.7E-6 in liver data set 1 and 2, respectively). Interestingly we also find a significant correlation between BMI and DNAm PhenoAge acceleration in the first adipose data set (r=0.43, p=1.2E-23 using n=648 adipose samples from the Twins UK study) but not in a second smaller adipose data set (n=32 samples).

DNAm PhenoAge and Immunosenescence

To test the hypothesis that DNAm PhenoAge captures aspects of the age-related decline of the immune system, we correlated DNAm PhenoAge with estimated[16, 18] blood cell count (**Supplementary Fig. S7**). After adjusting for age, we find that DNAm PhenoAgeAccel is negatively correlated with naïve CD8+ T cells (r=-0.35, p=9.2E-62), naïve CD4+ T cells (r=-0.29, p=4.2E-42), CD4+ helper T cells (r=-0.34, p=3.6E-58), and B cells (r=-0.18, p=8.4E-17). Further, DNAm PhenoAgeAccel is positively correlated with the proportion of granulocytes (r=0.32, p=2.3E-51), exhausted CD8+ (defined as CD28-CD45RA-) T cells (r=0.20, p=1.9E-20), and plasma blast cells (r=0.26, p=6.7E-34). These results are consistent with age related changes in blood cells[25] and suggest that DNAm PhenoAge may capture aspects of immunosenescence in blood. However, three lines of evidence suggest that DNAm PhenoAge is not simply a measure of immunosenescnce. First, another measure of immunosenescence, leukocyte telomere length, is only weakly correlated with DNAm PhenoAgeAccel (r=-0.087, P=7.6E-3) in the n=905 individuals from the Framingham Heart study, for whom both DNA methylation data and LTL data were available (**Supplementary Fig. S8**). Second, DNAm PhenoAge also applies to non-blood tissues. Third, the strong association between DNAm PhenoAge and mortality does not simply reflect changes in blood cell composition, as can be seen from the fact that in **Supplementary Fig. S9** the robust association remains even after adjusting for estimates of seven blood cell count measures (Meta(FE)=1.036, Meta p=5.6E-21).

Additional DNA sequence characteristics of the 513 CpGs in DNAm PhenoAge

The 513 CpGs in DNAm PhenoAge mapped to 506 distinct genes (**Table S5**). According to the GenAge database, 10 of the genes have implications for aging and/or longevity. These include: *CAT, CDKN1A, CSNK1E, INSR, HTRA2, NGFR, PMCH, PSEN1, SP1, UCP1*. Nevertheless, this does not signify a significant enrichment over what's expected by chance. Additionally, *ELOVL2*—for which there is growing evidence suggesting it harbors very promising DNAm age predictors[26-29]—is not part of our gene list, and in fact, the closest CpG is approximately 2,500k bp downstream.

The Genomic Regions Enrichment of Annotations Tool (GREAT)[30] was used to directly test for functional enrichment among the 506 genes. As shown in **Table S6**, CpGs in our score co-locate with genes enriched in the Kallikreins (KLK) gene family (Region Fold Enrichment=66.8, Binomial FDR=2.4E-4), as well as genes in the methylglyoxal degradation I pathway (Region Fold Enrichment=63.9, Binomial FDR=5.5E-3). However, in general, we find very little enrichment and as a result, we tested whether DNAm of each of the 513 CpGs was associated with differential expression in the gene that it co-located with. Based on transcriptional data from monocytes (n= 1,264) described in[23], our results show that, in general, very few of our CpGs are associated with changes in expression of the genes that they are supposedly linked to (**Table S7**), which may explain why this gene set produces generally null enrichment findings.

SUPPLEMENTARY TABLES

Supplementary Table S1: Phenotypic Aging Measures and Gompertz Coefficients. The table is analogous to Table 1 but includes the regression constant and gamma parameter of the Gompertz model.

Variable		Units	Weight
Albumin	Liver	g/L	-0.0336
Creatinine	Kidney	umol/L	0.0095
Glucose, serum	Metabolic	mmol/L	0.1953
C-reactive protein (log)	Inflammation	mg/dL	0.0954
Lymphocyte percent	Immune	%	-0.0120
Mean cell volume	Immune	fL	0.0268
Red cell distribution width	Immune	%	0.3306
Alkaline phosphatase	Liver	U/L	0.0019
White blood cell count	Immune	1000 cells/uL	0.0554
Age		Years	0.0804
Constant		-19.9067	
Gamma		0.0077	

Supplementary Table S2: Predictions of 10- and 20-Year Mortality Risk Based on Receiver Operating Characteristics Curves

Mortality Risk	Predictor Variable	ROC (AUC)	Std. Err.	Bonferroni P-Value
10 Year	DNAm PhenoAge	0.6177	0.0204	(Reference)
	Horvath DNAm Age	0.5605	0.0212	0.0185
	Hannum DNAm Age	0.5670	0.0216	0.04
20 Year	DNAm PhenoAge	0.5615	0.0132	(Reference)
	Horvath DNAm Age	0.5038	0.0133	0.0001
	Hannum DNAm Age	0.5228	0.0133	0.0089

Supplementary Table S3: Proportional Hazard Model (Gompertz) with All Three Epigenetic Aging Measures

	Hazard Ratio	SE	Z	P-Value	95% CI	
Chronological Age	1.078	0.010	8.420	<2.0E-16	1.059	1.097
DNAm PhenoAge	1.038	0.007	5.730	1.010E-08	1.025	1.051
Horvath DNAm Age	0.982	0.008	-2.220	2.600E-02	0.966	0.998
Hannum DNAm Age	1.006	0.009	0.700	4.850E-01	0.989	1.024
Gamma=3.48E-04						
Person Days= 12,917,217						
Deaths=757						
Log Likelihood=-1558.73						

Supplementary Table S4: Morbidity Associations Stratified by Smoking

	Comorbidity		Disease Free		CHD Risk		Physical Functioning	
**Sample**	**Coefficient**	**P-value**	**Coefficient**	**P-value**	**Coefficient**	**P-value**	**Coefficient**	**P-value**
**SMOKERS**								
WHI BA23 CurrentSmoker	0.013	3.41E-01	0.003	5.67E-01	0.007	6.67E-01	-0.794	1.23E-03
WHI BA23 FormerSmoker	0.021	5.04E-03	-0.006	2.53E-02	0.022	3.51E-02	-0.416	7.38E-04
WHI EMPC CurrentSmoker	0.032	2.39E-02	-0.025	3.50E-01	0.007	8.48E-01	-0.552	2.96E-02
WHI EMPC FormerSmoker	0.022	7.36E-04	-0.031	9.11E-03	0.039	6.95E-02	-0.451	1.13E-05
FHS CurrentSmoker	0.014	4.57E-01	-0.067	8.10E-02	0.046	3.35E-01	-0.116	1.85E-01
FHS FormerSmoker	0.021	2.71E-04	-0.024	1.12E-01	0.025	1.09E-03	-0.053	5.04E-02
NAS Smoking	0.027	2.73E-05	-0.076	4.02E-04	0.031	6.23E-02	NA	NA
JHS CurrentSmoker	0.014	1.13E-01	-0.018	4.74E-01	0.042	4.09E-01	NA	NA
JHS FormerSmoker	0.010	1.99E-01	-0.012	6.21E-01	0.033	1.98E-01	NA	NA
**Meta P-value (Stouffer)**		**3.74E-12**		**9.39E-06**		**2.13E-05**		**1.84E-11**
**NON-SMOKERS**								
WHI BA23 NonSmoker	0.010	7.73E-02	-0.003	1.30E-01	0.018	2.29E-02	-0.306	1.41E-03
WHI EMPC NonSmoker	0.019	1.07E-03	-0.016	1.19E-01	0.041	2.37E-02	-0.302	1.58E-03
FHS NonSmoker	0.016	3.76E-02	-0.043	1.50E-02	0.025	9.45E-02	0.044	2.53E-01
NAS NonSmoking	0.017	6.84E-02	-0.039	1.28E-01	0.029	1.99E-01	NA	NA
JHS NonSmoker	0.022	2.15E-08	-0.052	1.69E-05	0.027	2.68E-01	NA	NA
**Meta P-value (Stouffer)**		**7.92E-11**		**4.01E-07**		**1.20E-04**		**2.63E-03**

All models were adjusted for chronological age. Models for smokers were adjusted for pack-years. Models in all samples aside from WHI were adjusted for sex. Models in both WHI samples and NAS were adjusted for race/ethnicity.

Supplementary Table S5. Comparison of DNAm based epigenetic biomarkers of ageing

In the following pages, we provide a direct comparisons of 6 DNAm based biomarkers of ageing.

1. Multi-tissue DNAm age estimator based on 353 CpGs (Horvath 2013) [31]
2. DNA age estimator for blood based on 71 CpGs (Hannum et al 2013) [32]
3. Our new estimator DNAm PhenoAge estimator based on 513 CpGs (Levine et al 2018) [33]
4. DNAm Age estimator for blood based on 3 CpGs described in (Weidner et al 2014) [34]
5. Another version of the 3 CpG based DNAm Age estimator for blood (Weidner et al 2014) that was described by the same authors in Lin et al 2016 [35]
6. DNAm Age estimator for blood based on 99 CpGs (Lin et al 2016) [35].

We evaluate these predictors in terms of predicting time to death, relationship to body mass index, relationship to blood cell count estimates, telomere length, predicting centenarian status, neuritique plaques in postmortem brain samples.

Supplementary Table S5A. Predicting time to death based on epigenetic age acceleration in blood

Our comparison is mainly based the Illumina EPIC array data from n= 1756 African American individuals from the Jackson Heart study.

We evaluate whether DNAm age predict time to death. During the following up time, 282 passed away. The blood samples were collected at the baseline of the study (visit 1). At the time of the blood draw, the individuals ranged from 22 to 93 (median age 57). At the time of the last follow up, 282 individuals were known to be deceased. The median number of years of follow up (time to death or last follow up) was 12.2 years (ranging from 0.14 to 14.5 years).

In the following, we report the results of this Cox regression analysis which demonstrate that DNAmPhenoAge has the most predictive association with time to death.

The DNAmAge estimator is listed in the heading.

```
[1] Covariate: "DNAmAge Horvath 2013"

  n= 1756, number of events= 282

                   coef exp(coef) se(coef)     z Pr(>|z|)
Age             0.06553   1.06772  0.01058 6.194 5.85e-10 ***
DNAmBiomarker 0.03573   1.03638  0.01267 2.820   0.0048 **

                exp(coef) exp(-coef) lower .95 upper .95
Age                 1.068     0.9366     1.046     1.090
DNAmBiomarker     1.036     0.9649     1.011     1.062

Concordance= 0.772  (se = 0.018 )

Rsquare= 0.15   (max possible= 0.903 )


[1] "DNAmAge Hannum 2013"

                   coef exp(coef) se(coef)     z Pr(>|z|)
Age             0.03770   1.03842  0.01137 3.315 0.000917 ***
DNAmBiomarker 0.06905   1.07149  0.01263 5.466  4.6e-08 ***

                exp(coef) exp(-coef) lower .95 upper .95
Age                 1.038     0.9630     1.016     1.062
```

```
DNAmBiomarker     1.071     0.9333     1.045     1.098
Concordance= 0.779  (se = 0.018 )
Rsquare= 0.159   (max possible= 0.903 )

[1] "DNAm PhenoAge"
                    coef exp(coef) se(coef)     z Pr(>|z|)
Age             0.038863  1.039628 0.009323 4.168 3.07e-05 ***
DNAmBiomarker 0.060463  1.062329 0.008456 7.151 8.64e-13 ***
                exp(coef) exp(-coef) lower .95 upper .95
Age                 1.040     0.9619     1.021     1.059
DNAmBiomarker     1.062     0.9413     1.045     1.080
Concordance= 0.789  (se = 0.018 )
Rsquare= 0.168   (max possible= 0.903 )
[1] "DNAmAge Weidner"
                     coef exp(coef)  se(coef)      z Pr(>|z|)
Age              0.091318  1.095618  0.006788 13.453   <2e-16 ***
DNAmBiomarker -0.002031  0.997972  0.006871 -0.296    0.768
                exp(coef) exp(-coef) lower .95 upper .95
Age                 1.096     0.9127    1.0811     1.110
DNAmBiomarker     0.998     1.0020    0.9846     1.012

Concordance= 0.768  (se = 0.018 )
Rsquare= 0.146   (max possible= 0.903 )
[1] "DNAmAgeWeidnerVersion2"
                    coef exp(coef) se(coef)      z Pr(>|z|)
Age             0.087910  1.091890 0.007196 12.217   <2e-16 ***
DNAmBiomarker 0.005020  1.005033 0.009035  0.556    0.578
                exp(coef) exp(-coef) lower .95 upper .95
Age                 1.092     0.9158    1.0766     1.107
DNAmBiomarker     1.005     0.9950    0.9874     1.023
Concordance= 0.768  (se = 0.018 )
Rsquare= 0.146   (max possible= 0.903 )
 [1] "DNAmAgeLinWagner"
                    coef exp(coef) se(coef)     z Pr(>|z|)
Age             0.062002  1.063965 0.009885 6.272 3.56e-10 ***
DNAmBiomarker 0.034258  1.034852 0.009617 3.562 0.000367 ***

                exp(coef) exp(-coef) lower .95 upper .95
Age                 1.064     0.9399     1.044     1.085
DNAmBiomarker     1.035     0.9663     1.016     1.055
```

```
Concordance= 0.773  (se = 0.018 )
Rsquare= 0.152   (max possible= 0.903 )
```

Supplementary Table S5B. Body Mass Index versus epigenetic age acceleration in blood

Using the EPIC DNA methylation data from blood samples from the Jackson Heart study we regressed DNAmAge (dependent variable) on age, BMI, and gender.

```
[1] "DNAmAge Horvath 2013"
               Estimate  Std. Error   t value     Pr(>|t|)
(Intercept)  8.81156753 0.725692568 12.142287 1.242994e-32
Age          0.75525714 0.008641536 87.398485 0.000000e+00
BMI          0.05384969 0.014626637  3.681618 2.387880e-04
Female      -1.50753799 0.221553035 -6.804411 1.388753e-11
[1] "DNAmAge Hannum 2013"
               Estimate  Std. Error    t value     Pr(>|t|)
(Intercept) -3.26768612 0.665873027  -4.907371 1.008795e-06
Age          0.79985115 0.007929206 100.874059 0.000000e+00
BMI          0.06136597 0.013420949   4.572402 5.159818e-06
Female      -1.48772717 0.203290204  -7.318243 3.810522e-13
[1] "DNAm PhenoAge"
              Estimate Std. Error   t value     Pr(>|t|)
(Intercept) -9.6092491 0.99209686 -9.685797 1.197457e-21
Age          0.8798796 0.01181387 74.478502 0.000000e+00
BMI          0.1387421 0.01999613  6.938446 5.561863e-12
Female      -0.8038841 0.30288593 -2.654082 8.024474e-03
 [1] "DNAmAge Weidner 2014"
               Estimate Std. Error    t value      Pr(>|t|)
(Intercept) 14.69188745 1.39338191 10.5440492  3.037441e-25
Age          0.53816312 0.01659237 32.4343732 4.148625e-181
BMI          0.01954192 0.02808420  0.6958335  4.866255e-01
Female       0.27490420 0.42539776  0.6462286  5.182160e-01
[1] "DNAmAge WeidnerVersion2"
               Estimate Std. Error    t value      Pr(>|t|)
(Intercept) 27.73994403 0.98879353 28.0543341 2.541079e-143
Age          0.44024067 0.01177454 37.3892098 2.187252e-225
BMI          0.04365288 0.01992955  2.1903596  2.862971e-02
Female      -0.21365122 0.30187743 -0.7077416  4.792000e-01
[1] "DNAmAge LinWagner"
                Estimate Std. Error    t value      Pr(>|t|)
```

```
(Intercept) -15.65943376 0.94417454 -16.585317 1.718059e-57
Age           0.86028517 0.01124322  76.515936 0.000000e+00
BMI           0.07756353 0.01903023   4.075806 4.790863e-05
Female       -0.96694177 0.28825531  -3.354463 8.121766e-04
```

Supplementary Table S5C. Leukocyte telomere length versus epigenetic age acceleration

We used Illumina EPIC DNA methylation data from blood samples of the Jackson Heart Study to test the association between the DNAm age estimators (dependent variable) and Leukocyte Telomere Length (more precisely Mean Telomere Restriction Fragment) using multivariate regression models that also included chronological age and sex as covariates.

The following analysis reveals that DNAmPhenoAge has the strongest association with leukocyte telomere length.

```
[1] Dependant variable "DNAmAge Horvath2013"
              Estimate  Std. Error   t value     Pr(>|t|)
(Intercept) 12.1545948 1.581513271  7.685421 2.613873e-14
Age          0.7492805 0.009916796 75.556707 0.000000e+00
Mean.TRF    -0.1862622 0.179023240 -1.040436 2.982913e-01
Female      -1.3774774 0.230804282 -5.968162 2.936492e-09
[1] "DNAmAge Hannum 2013"
              Estimate  Std. Error   t value     Pr(>|t|)
(Intercept)  4.1633323 1.429829789  2.911768 3.642645e-03
Age          0.7782609 0.008965673 86.804516 0.000000e+00
Mean.TRF    -0.6177930 0.161853059 -3.816999 1.401240e-04
Female      -1.2028016 0.208667764 -5.764195 9.789582e-09
[1] "DNAm PhenoAge"
              Estimate Std. Error    t value     Pr(>|t|)
(Intercept)  5.3155827 2.16085288  2.4599466 1.399866e-02
Age          0.8410257 0.01354951 62.0705366 0.000000e+00
Mean.TRF    -1.2129200 0.24460299 -4.9587291 7.833534e-07
Female      -0.2312638 0.31535246 -0.7333504 4.634500e-01
 [1] "DNAmAge Weidner 2014"
              Estimate Std. Error   t value      Pr(>|t|)
(Intercept) 24.3774102 2.99735672  8.132969  8.199171e-16
Age          0.5087108 0.01879477 27.066615 1.306044e-133
Mean.TRF    -1.0583315 0.33929308 -3.119225  1.844941e-03
Female       0.6614184 0.43743090  1.512052  1.307139e-01
[1] "DNAmAgeWeidnerVersion2"
               Estimate Std. Error    t value      Pr(>|t|)
(Intercept) 33.36899186  2.1108708 15.8081643  1.726666e-52
Age          0.42149161  0.0132361 31.8440823 1.585201e-173
```

```
Mean.TRF    -0.47549009  0.2389451 -1.9899550  4.676231e-02
Female       0.08632544  0.3080581  0.2802245  7.793407e-01
[1] "DNAmAgeLinWagner"
              Estimate Std. Error   t value     Pr(>|t|)
(Intercept) -9.8222427 2.04436602 -4.804542 1.693176e-06
Age          0.8439815 0.01281909 65.837863 0.000000e+00
Mean.TRF    -0.3591097 0.23141698 -1.551786 1.209070e-01
Female      -0.7183775 0.29835250 -2.407815 1.615853e-02
```

Supplementary Table S5D. Current Smoker status versus epigenetic age acceleration in blood

We regressed DNAm age on current smoker status in the Jackson Heart Study data.

```
 [1] "DNAmAge Horvath 2013"
                 Estimate  Std. Error    t value     Pr(>|t|)
(Intercept)   10.66505021 0.519203735 20.5411662 4.201095e-84
Age            0.75211487 0.008638039 87.0700928 0.000000e+00
currentSmoker -0.08919795 0.307986571 -0.2896164 7.721444e-01
Female        -1.37481629 0.221213224 -6.2148920 6.417278e-10
[1] "DNAmAge Hannum 2013"
                Estimate  Std. Error    t value     Pr(>|t|)
(Intercept)   -1.3736921 0.475312443  -2.890082 3.899275e-03
Age            0.7965832 0.007907816 100.733653 0.000000e+00
currentSmoker  0.8861828 0.281950687   3.143042 1.700297e-03
Female        -1.2499419 0.202512792  -6.172163 8.367406e-10
[1] "DNAm PhenoAge"
                Estimate Std. Error    t value     Pr(>|t|)
(Intercept)   -5.4308204 0.71140186 -7.6339700 3.734606e-14
Age            0.8725501 0.01183566 73.7221513 0.000000e+00
currentSmoker  2.3696247 0.42199662  5.6152693 2.281171e-08
Female        -0.2372610 0.30310163 -0.7827769 4.338649e-01
 [1] "DNAmAge Weidner 2014"
                Estimate Std. Error   t value      Pr(>|t|)
(Intercept)   14.7588297 0.98899296 14.923089  1.799328e-47
Age            0.5427897 0.01645396 32.988385 1.143641e-185
currentSmoker  1.6157787 0.58666094  2.754195  5.945074e-03
Female         0.4523669 0.42137278  1.073555  2.831714e-01
[1] "DNAmAge WeidnerVersion2"
                Estimate Std. Error    t value      Pr(>|t|)
(Intercept)   29.4805264  0.7032600 41.9198121 7.539043e-266
Age            0.4357210  0.0117002 37.2404766 1.404731e-223
currentSmoker -0.7641549  0.4171669 -1.8317725  6.715668e-02
```

```
Female          -0.1493001  0.2996327 -0.4982771  6.183519e-01
[1] "DNAmAge LinWagner"
                 Estimate Std. Error     t value     Pr(>|t|)
(Intercept)    -12.8628901 0.67757323 -18.9837638 3.626725e-73
Age              0.8534952 0.01127285  75.7124787 0.000000e+00
currentSmoker   -0.1575977 0.40192980  -0.3921025 6.950306e-01
Female          -0.7985192 0.28868852  -2.7660233 5.734593e-03
```

<u>Supplementary Table S5E. Offspring of centenarians have low epigenetic age acceleration in blood</u>

We analyzed Illumina 450K data from Italian semi-supercentenarians (individuals aged 105 or more), their offspring and age matched controls from [36]. We analyzed peripheral blood mononuclear cells from Italian families constituted of 82 semi-supercentenarians (mean age: 105.6 ± 1.6 years), 63 semi-supercentenarians' offspring (mean age: 71.8 ± 7.8 years), and 47 age-matched controls (mean age: 69.8 ± 7.2 years). Using a linear regression model, we demonstrate that the offspring of semi-supercentenarians have a lower epigenetic age than age-matched controls [36].

In R language lm(DNAmAge~Age+Status, data=datSample, subset=Age<100)).

The negative sign of the Student T statistic indicates that the offspring of centenerians are younger than age matched controls.

```
[1] Dependant variable: "DNAmAge Horvath 2013"
                Estimate Std. Error t value Pr(>|t|)
(Intercept)        12.00      5.200     2.4  1.9e-02
Age                 0.82      0.073    11.0  6.7e-20
StatusOffspring    -3.00      1.100    -2.7  8.1e-03

[1] Dependant variable: "DNAmAgeHannum"
                Estimate Std. Error t value Pr(>|t|)
(Intercept)        16.00      5.400     3.0  3.5e-03
Age                 0.78      0.076    10.0  1.5e-17
StatusOffspring    -1.80      1.200    -1.6  1.2e-01

[1] "DNAmPhenoAge"
                Estimate Std. Error t value Pr(>|t|)
(Intercept)         7.50      6.100     1.2  2.2e-01
Age                 0.73      0.085     8.6  8.3e-14
StatusOffspring    -2.40      1.300    -1.9  6.5e-02

 [1] "DNAmAge Weidner 2014"
                Estimate Std. Error t value Pr(>|t|)
```

```
(Intercept)          8.80        9.80    0.90  3.7e-01
Age                  0.74        0.14    5.40  4.5e-07
StatusOffspring     -0.25        2.10   -0.12  9.1e-01

[1] "DNAmAge WeidnerVersion2"
                Estimate Std. Error t value Pr(>|t|)
(Intercept)          35.0       6.400     5.5  3.1e-07
Age                   0.5       0.091     5.5  2.8e-07
StatusOffspring      -1.7       1.400    -1.2  2.3e-01

[1] "DNAmAge LinWagner"
                Estimate Std. Error t value Pr(>|t|)
(Intercept)         -0.62       6.000   -0.10  9.2e-01
Age                  0.79       0.085    9.30  1.9e-15
StatusOffspring     -0.21       1.300   -0.16  8.7e-01
```

Supplementary Table S5F. **Neuritique plaques vs age acceleration in the prefrontal cortex**

We used the DNA methylation data from postmortem prefrontal cortex samples of the Religious Order Study and the MAP study [37]. We correlated measures of age acceleration in the prefrontal cortex with the abundance of neuritique plaques (adjusted for chronological age and gender).

	Cor with age adjusted neuritique plaques
AAHorvath	0.13841331
AAHannum	0.13489693
AAPheno	0.09474522
AAWeidner	0.05201257
AA.WeidnerVersion2	0.06572580
AA.LinWagner	0.12125363

Supplementary Table S5F. **Body Mass Index versus epigenetic age acceleration in liver**

We used n=85 Illumina 450K data arrays from human liver samples. Liver samples from morbidly obese patients with all stages of NAFLD and controls were analysed. The data are described in [24, 38] and available from Gene Expression Omnibus (GSE48325).

Using multivariate linear regression models (dependent variable DNAm Age) we find that high BMI is associated with increased DNAm Age even after correcting for gender and age consistent with the findings reported in [39].

```
[1] Dependant variable: "DNAmAge"
```

```
            Estimate Std. Error t value Pr(>|t|)
(Intercept)     5.70      3.200     1.8  7.8e-02
Age             0.75      0.042    18.0  5.5e-30
BMI             0.15      0.042     3.7  4.2e-04
Female         -0.60      1.200    -0.5  6.2e-01
[1] "DNAmAgeHannum"
            Estimate Std. Error t value Pr(>|t|)
(Intercept)   28.000      3.000     9.3  2.0e-14
Age            0.720      0.039    18.0  1.5e-30
BMI            0.091      0.039     2.3  2.4e-02
Female         1.500      1.100     1.3  1.9e-01
[1] "DNAmPhenoAge"
            Estimate Std. Error t value Pr(>|t|)
(Intercept)    -4.50      3.900   -1.20  2.5e-01
Age             0.64      0.050   13.00  5.3e-21
BMI             0.13      0.051    2.60  1.0e-02
Female         -0.81      1.400   -0.56  5.7e-01
 [1] "DNAmAgeWeidner"
            Estimate Std. Error t value Pr(>|t|)
(Intercept)    24.00      4.000     6.0  4.4e-08
Age             0.58      0.052    11.0  5.5e-18
BMI             0.12      0.053     2.3  2.6e-02
Female         -2.00      1.500    -1.3  1.8e-01
[1] "DNAmAgeWeidnerVersion2"
            Estimate Std. Error t value Pr(>|t|)
(Intercept)   77.000      3.200   24.00  2.4e-38
Age            0.230      0.042    5.50  4.9e-07
BMI           -0.018      0.042   -0.44  6.6e-01
Female        -0.640      1.200   -0.54  5.9e-01
[1] "DNAmAgeLinWagner"
            Estimate Std. Error t value Pr(>|t|)
(Intercept)    41.00      6.600     6.2  2.2e-08
Age             0.79      0.086     9.1  4.2e-14
BMI             0.15      0.086     1.7  9.5e-02
Female          3.80      2.400     1.6  1.2e-01
```

Supplementary Table S5G. **Naïve CD8+T cell and Granulocyte Cell abundance versus epigenetic age acceleration in blood**

We use Illumina 450K DNA methylation data from blood samples from over 2k postmenopausal women from the Women's Health Initiative [18]. We estimated blood cell proportions/counts using two different software tools. Houseman's estimation method [16] was used to estimate the proportions of cytotoxic (CD8+) T cells, helper (CD4+) T, natural killer, B cells, and granulocytes (mostly neutrophils). Another method was used to estimate the percentage of exhausted CD8+ T cells (defined as CD28-CD45RA-), plasmablasts, and the number (count) of naïve CD8+ T cells (defined as CD45RA+CCR7+) [40], which is implemented in the advanced analysis option of the epigenetic age calculator software [41]. Imputed blood cell counts have moderately high correlations with corresponding flow cytometric data [18]. All blood cell counts were adjusted for chronolgical age, i.e. the reported correlations are not confounded by chronological age.

Correlation Table						
	Horvath 353	Hannum 71	PhenoAge	Weidner	WeidnerVer	99 LinWagner
PlasmaBlastAdjAge	-0.02	0.14	0.19	-0.28	-0.06	0.02
CD8pCD28nCD45RAnAdjAg	0.14	0.21	0.16	0.13	0.13	0.11
CD8.naiveAdjAge	-0.11	-0.24	-0.25	-0.15	-0.06	-0.09
CD4.naiveAdjAge	-0.04	-0.18	-0.23	-0.07	0.05	-0.08
CD8TAdjAge	0.16	0.03	-0.10	0.51	0.26	0.09
CD4TAdjAge	-0.13	-0.24	-0.26	-0.04	-0.18	-0.10
NKAdjAge	0.07	0.08	-0.05	0.27	-0.01	0.07
BcellAdjAge	-0.11	-0.11	-0.13	0.11	-0.14	0.04
MonoAdjAge	-0.01	0.00	0.02	-0.05	-0.01	0.01
GranAdjAge	-0.01	0.14	0.25	-0.33	-0.05	0.01

Supplementary Table S6: Characteristics of the 513 CpGs

[Separate PDF]

Supplementary Table S7: Associations between DNAm and expression for CpG-Gene pairs

[Separate excel file]

Supplementary Table S8: GO for differentially expressed genes as a function of DNAm PhenoAgeAccel

[Separate excel file]

Supplementary Table S9: Comparison with Zhang DNAm Mortality Scores

	Zhang Score		DNAm PhenoAge	
	HR (95% CI)	P-value	HR (95% CI)	P-value
**Age Adjusted Models**				
**Categorical Variable (Reference= Category 1)**				
Category 2	1.18 (0.95-1.48)	1.42E-1	1.21 (0.92-1.58)	1.68E-1
Category 3	1.49 (1.21-1.83)	1.63E-4	1.80 (1.36-2.38)	3.25E-5
Category 4	2.25 (1.34-3.76)	2.12E-3	2.90 (1.75-4.80)	3.41E-5
Pseudo R-squared	0.096		.099	
**Standardized Continuous Variable**	1.27 (1.17-1.39)	5.19E-8	1.45 (1.30-1.62)	4.48E-11
Pseudo R-squared	0.113		0.105	
**Fully Adjusted Models**				
**Categorical Variable (Reference= Category 1)**				
Category 2	1.11 (0.89-1.39)	3.67E-1	1.21 (0.92-1.58)	1.66E-1
Category 3	1.31 (1.06-1.63)	1.22E-2	1.67 (1.27-2.21)	2.89E-4
Category 4	1.92 (1.14-3.23)	1.48E-2	2.62 (1.57-4.40)	2.32E-4
Pseudo R-squared	0.110		.114	
**Standardized Continuous Variable**	1.20 (1.10-1.32)	3.80E-5	1.40 (1.25-1.57)	4.10E-9
Pseudo R-squared	0.113		0.120	

Results come from cox proportional hazard models for all-cause mortality in the WHI (BAA23 sample with up to ~20 years of mortality follow-up). Fully adjusted models included chronological age, race/ethnicity, and smoking status. Results for continuous predictors represent the hazard ratio for a one standard deviation increase in each DNAm variable. Our sample had a total of 559 deaths and 31,025.21 person-years.

Supplementary Table S10: Correlations between DNAm Scores (adjusted for age)

	DNAm PhenoAgeAccel	Horvath DNAm AgeAccel	Hannum DNAM AgeAccel
DNAm PhenoAgeAccel	1		
Horvath DNAm AgeAccel	0.46	1	
Hannum DNAM AgeAccel	0.482	0.511	1

SUPPLEMENTARY FIGURES

SUPPLEMENTARY FIG. S1. Association between phenotypic age and morbidity.

Data on n=6,209 participants from NHANES IV show that phenotypic age (adjusted for chronological age) is associated with A) a person's number of coexisting morbidities and B) physical functioning problems in a strong dose-dependent manner. Results are based on Kruskal Wallis test.

A

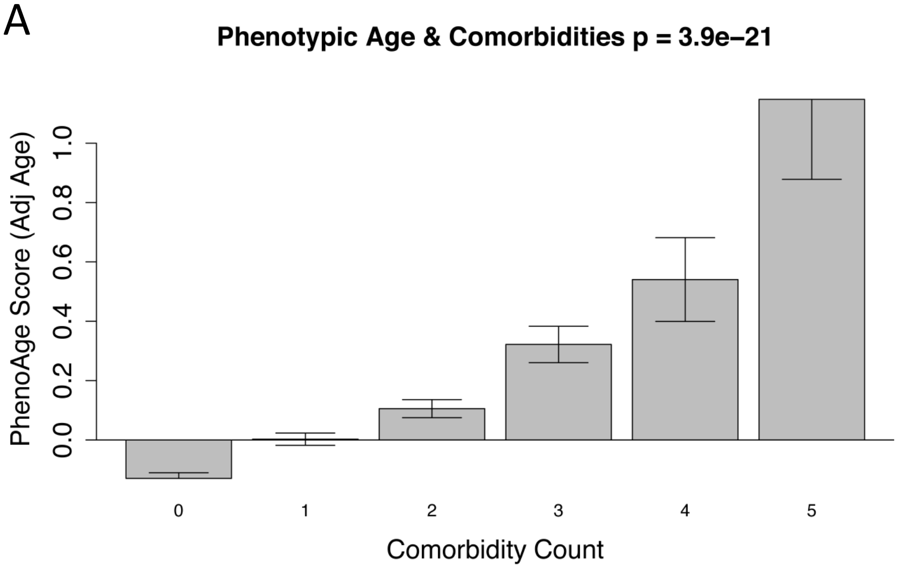

B

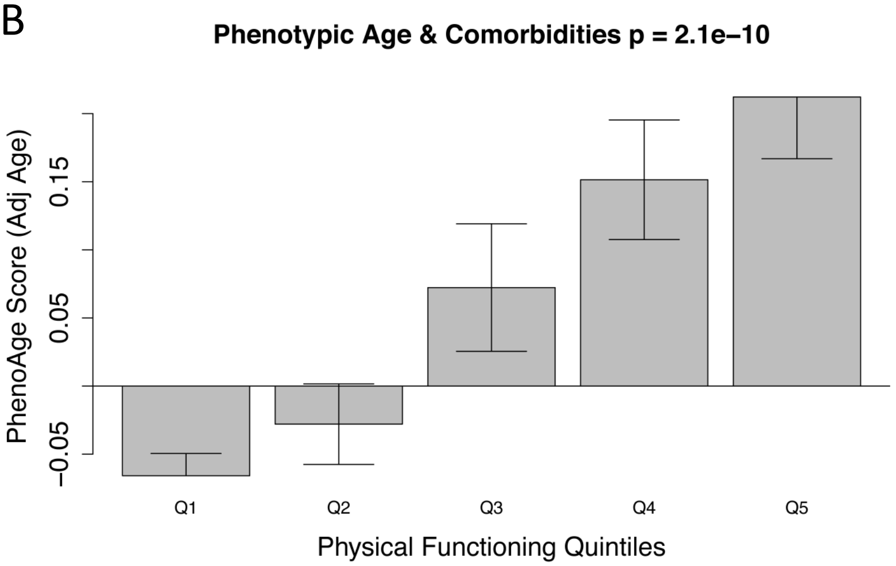

SUPPLEMENTARY FIG. S2. Longitudinal comparisons of phenotypic age and DNAm PhenoAge.

Over the nine years of follow-up, mean and median change in phenotypic age (A) and DNAm PhenoAge (B) was about 9 years. Nevertheless, within person (age adjusted) measures for phenotypic age (C) and DNAm PhenoAge (D), remained fairly stable over time—those who are fast agers, remain fast agers. Finally, panel E shows the correlation between change in phenotypic age and change in DNAm PhenoAge, suggesting that those who experience an acceleration of phenotypic age based on clinical markers also experience age acceleration on an epigenetic level.

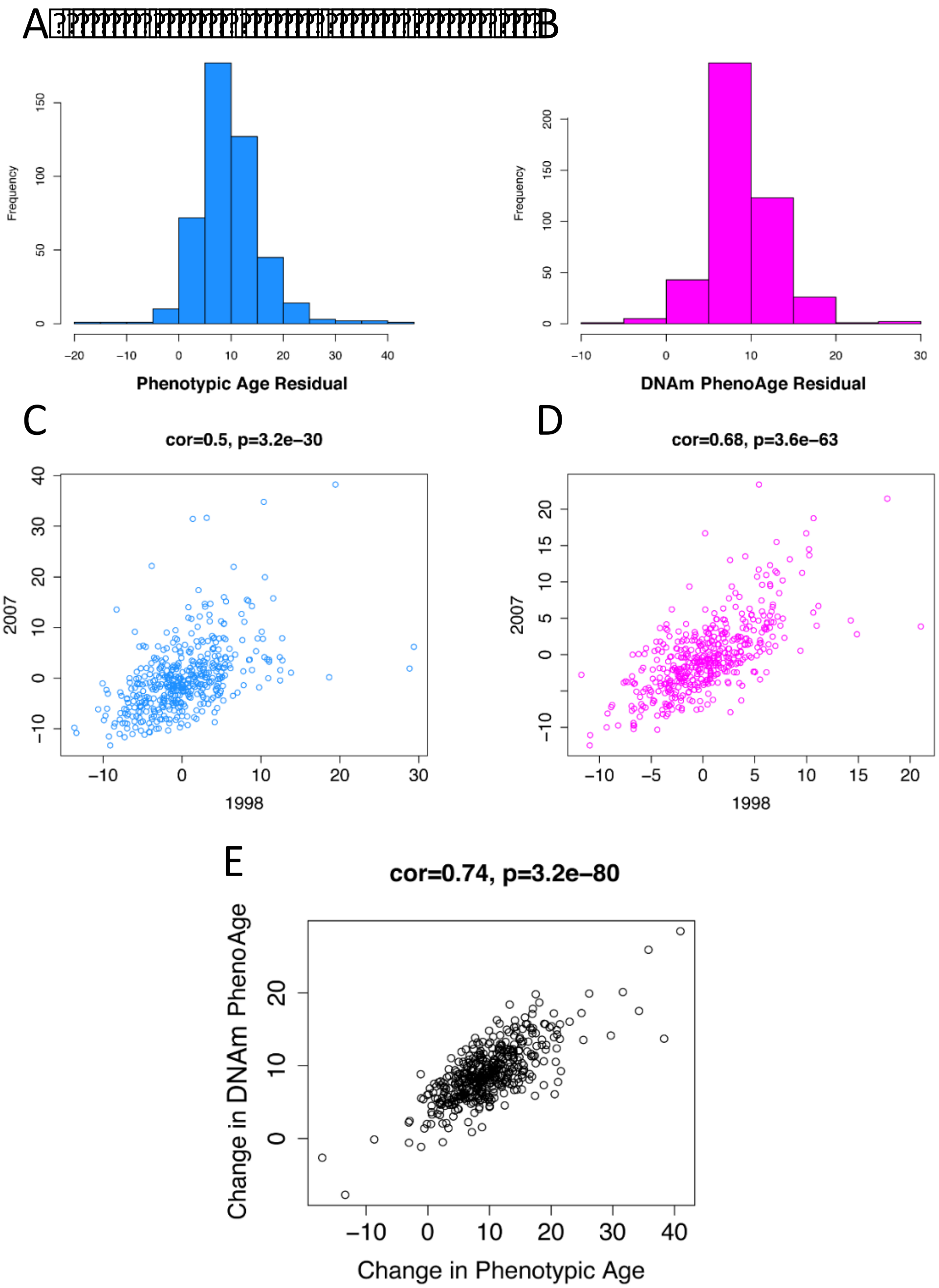

SUPPLEMENTARY FIG. S3. Associations between smoking and DNAm PhenoAge.

When comparing DNAm PhenoAge by smoking status, we find that current smokers have significantly higher epigenetic ages compared to never, and/or former smokers (A). This is also true when comparing DNAm PhenoAge as a function of pack-years (B). However, no associations with pack-years are found when stratifying by smoking status—current (D) or former (D)—suggesting that the correlation with pack-years in (B) merely reflects a difference in smoking status (similar to what is shown in A).

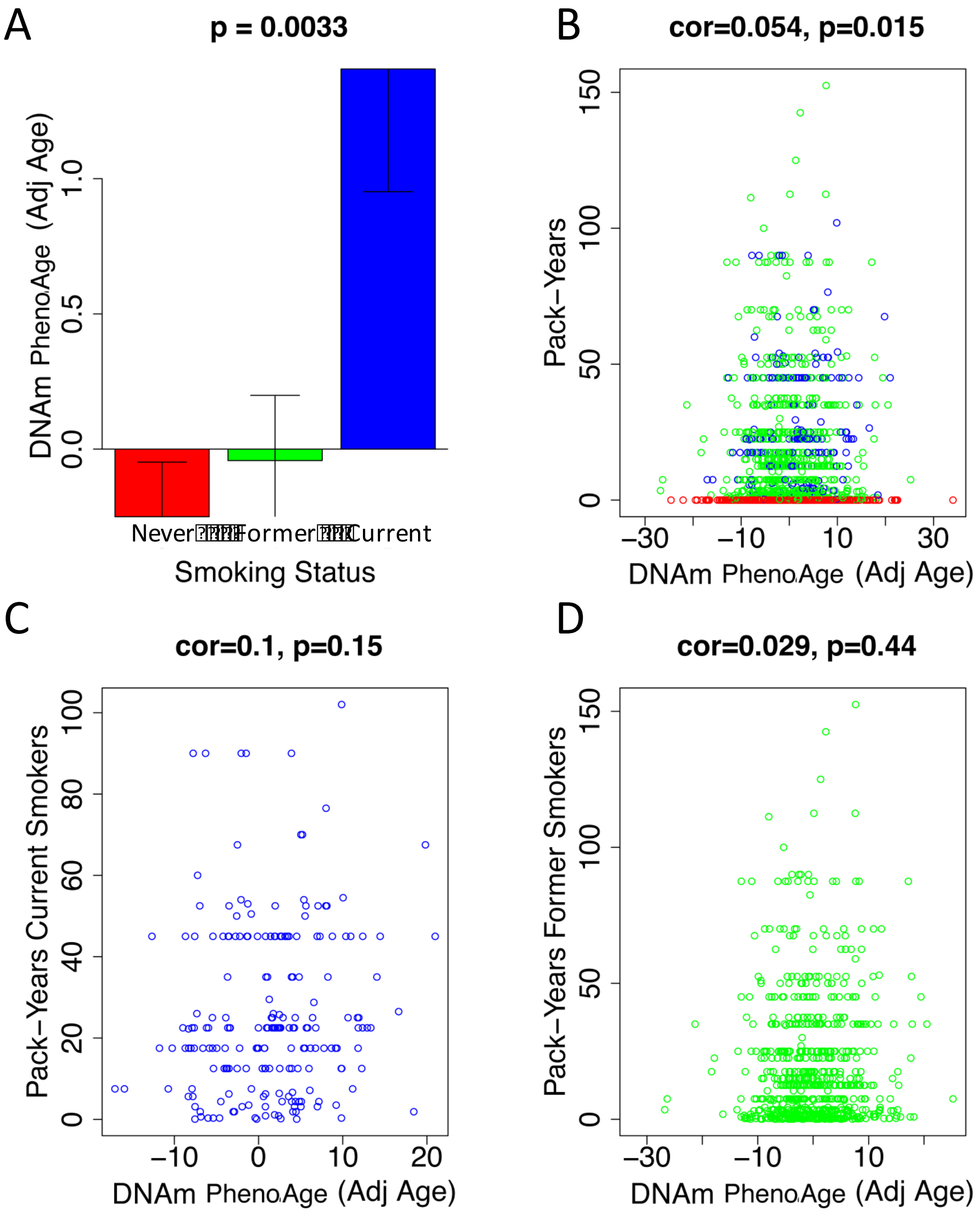

SUPPLEMENTARY FIG. S4. Fixed effect meta-analysis of the effect of DNAm PhenoAge on the hazard of all-cause mortality, stratifying by smoking. In smoking stratified analyses, adjusting for pack-years (in smokers) and chronological age, we find that DNAm PhenoAge significantly predicts mortality even within groups, and despite much smaller sample sizes (panel A depicts results for non-smokers and panel B depicts results for smokers). The Hannum measure also relates to mortality in both smokers and non-smokers; although to a lesser degree than DNAm PhenoAge. Interestingly, the effect of DNAm PhenoAge on mortality appears to be larger for smokers, compared to non-smokers, suggesting that it may capture vulnerability to stressors, like cigarette exposure.

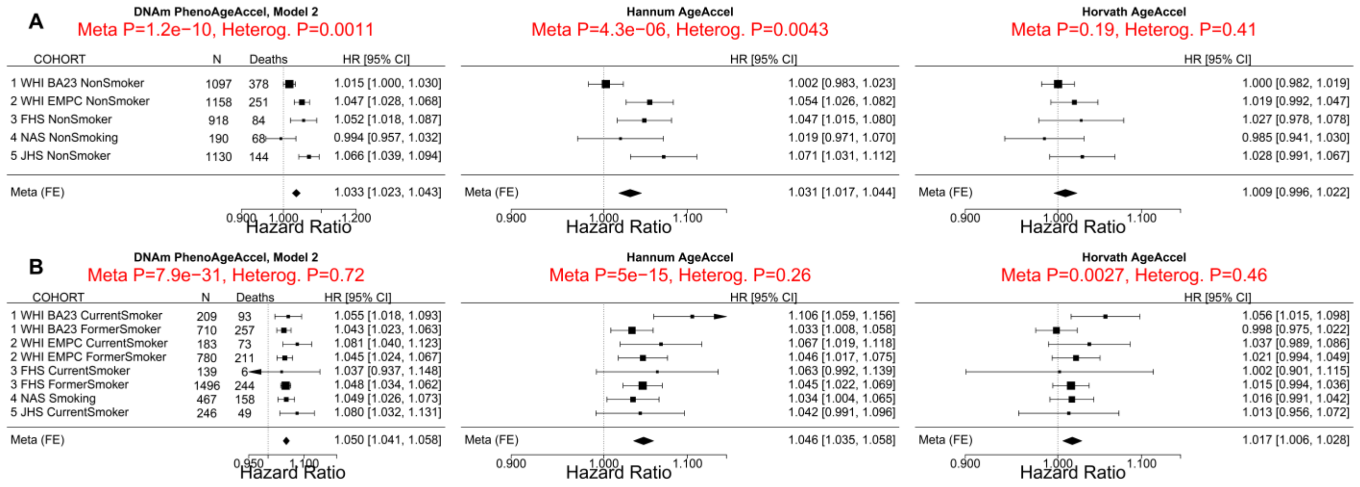

SUPPLEMENTARY FIG. S5. Associations between DNAm PhenoAge and race/ethnicity in the WHI.

When comparing DNAm PhenoAge by race/ethnicity, we find that non-Hispanic blacks have the highest DNAm PhenoAges, whereas non-Hispanic whites have the lowest (A). This is reflective of trends we see in life-expectancy. Further, this likely reflects differences between the three groups, rather than variations in the reliability of the measure within the three strata, as evidenced by the very consistent age trends across all three groups (B, C, & D).

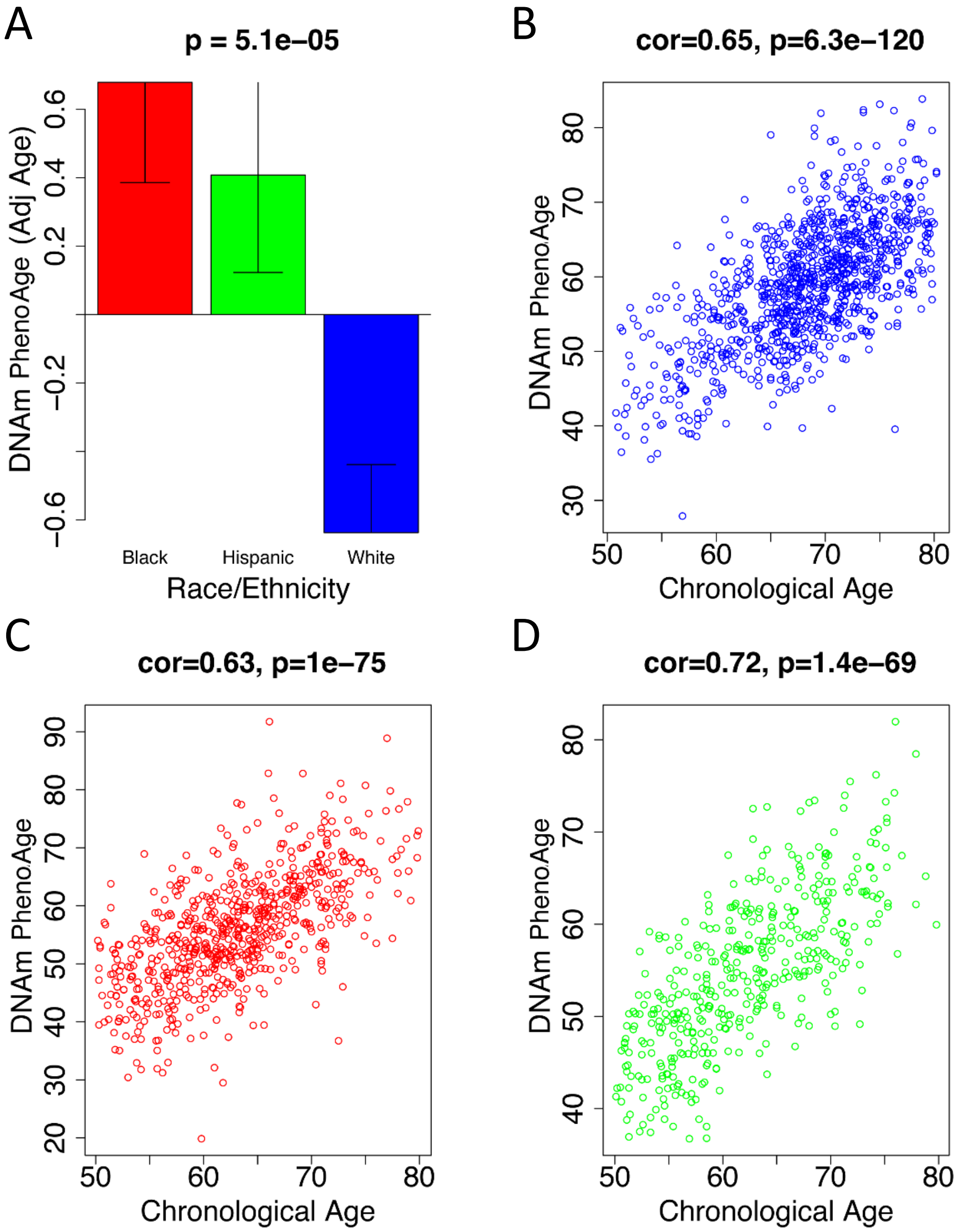

SUPPLEMENTARY FIG. S6. Associations with measures of age acceleration in the WHI. Correlations (bicor, biweight midcorrelation) between select variables and DNAm PhenoAgeAccel (A) show that education, income, exercise, proxies of fruit/vegetable consumption, and HDL cholesterol are negatively associated (blue) with DNAm PhenoAge. Conversely, CRP, insulin, glucose, triglycerides, BMI, waist-to-hip ratio, and systolic blood pressure have a positive association (red) with DNAm PhenoAge. Similar results are found in multivariate regression models (B).

A) Correlations (marginal) with Outcome=DNAmPhenoAgeAccel

		Pooled WHI samples, Adjusted for ethnicity and dataset		PhenoAgeAccel	
		n	μ	bicor	p
Diet	log2(Total energy)	3700	10.58	0.01	0.40
	Carbohydrate	3700	48.64	-0.02	0.23
	Protein	3700	16.39	-0.01	0.45
	Fat	3700	35.05	0.03	0.12
	log2(1+Red meat)	3700	0.69	0.05	2E-3
	log2(1+Poultry)	3700	0.39	-0.02	0.14
	log2(1+Fish)	3700	0.25	-0.03	0.10
	log2(1+Dairy)	3700	1.21	-0.01	0.76
	log2(1+Whole grains)	3700	1.00	-0.02	0.19
	log2(1+Nuts)	3700	0.10	-0.04	0.03
	log2(Fruits)	3700	0.57	-0.02	0.13
	log2(Vegetables)	3700	0.71	-0.03	0.09
Dietary biomarkers	Retinol	2267	0.58	0.03	0.17
	Mean carotenoids	2266	0.06	-0.22	**2E-27**
	Lycopene	2267	0.36	-0.11	**3E-7**
	log2(alpha-Carotene)	2267	-4.21	-0.19	**5E-20**
	log2(beta-Carotene)	2266	-2.15	-0.18	**2E-17**
	log2(Lutein+Zeaxanthin)	2267	-2.38	-0.17	**2E-16**
	log2(beta-Cryptoxanthin)	2267	-3.76	-0.17	**2E-15**
	log2(alpha-Tocopherol)	2267	3.87	-0.03	0.19
	log2(gamma-Tocopherol)	2267	0.88	0.07	**6E-4**
Measurements	log2(C-reactive protein)	2809	1.57	0.18	**5E-22**
	log2(Insulin)	4042	5.78	0.15	**2E-20**
	log2(Glucose)	4144	6.57	0.10	**2E-10**
	log2(Triglyceride)	4148	7.02	0.09	**5E-9**
	Total cholesterol	4148	224.00	-0.04	4E-3
	LDL cholesterol	4084	140.00	-0.05	2E-3
	HDL cholesterol	4145	53.00	-0.09	**7E-9**
	log2(Creatinine)	2748	-0.43	0.01	0.52
	Systolic blood pressure	4177	129.00	0.08	**1E-6**
	Diastolic blood pressure	4178	76.00	0.02	0.12
	log2(Waist / hip ratio)	4037	-0.28	0.15	**5E-22**
	BMI	4145	28.88	0.13	**5E-16**
Socio-behavioral	Education	4143	7.00	-0.09	**6E-9**
	Income	4054	3.00	-0.06	**9E-5**
	log2(1+Exercise)	3914	2.75	-0.06	**7E-5**
	Current smoker	2321	0.00	0.10	**3E-6**
	log2(1+Alcohol)	3700	0.14	-0.04	0.02

B) Multivariate Lin. Models. DNAmPhenoAgeAccel

PhenoAgeAccel	Model 1		Model 2		Model 3		Model 4		Model 5	
	Minimal		*Food*		*Biomarkers*		*MetSyn*		*Full*	
n=2568	β	p	β	p	β	p	β	p	β	p
log(1 + Red meat)			2.91	**0.01**					2.07	0.05
log(1 + Poultry)			-0.75	0.65					-0.91	0.57
log(1 + Fish)			1.60	0.38					1.78	0.32
log(1 + Dairy)			-0.63	0.43					-0.54	0.49
log(1 + Nuts)			-3.93	**0.02**					-3.80	**0.02**
log(1 + Whole grains)			1.14	0.22					1.42	0.12
log(1 + Fruits)			0.30	0.43					0.33	0.37
log(1 + Vegetables)			-0.66	0.18					-0.55	0.26
Current drinker			-0.84	0.10					-0.61	0.23
African American	0.73	**0.02**	0.58	0.09	0.50	0.13	0.62	**0.05**	0.37	0.30
Hispanic	0.54	0.15	1.16	0.37	0.59	0.64	0.78	0.53	0.03	0.98
Education	-0.29	**2E-5**	-0.26	**1E-4**	-0.26	**1E-4**	-0.27	**4E-5**	-0.25	**3E-4**
BMI	0.14	**7E-10**	0.12	**5E-8**	0.02	0.54	0.01	0.60	0.01	0.68
Physically active	-0.02	0.14	-0.47	0.08	-0.29	0.26	-0.32	0.21	-0.26	0.33
Current smoker	1.67	**2E-4**	5.23	**5E-4**	4.35	**3E-3**	4.18	**4E-3**	4.30	**4E-3**
log(C-reactive protein)					2.09	**5E-11**	2.18	**3E-12**	2.10	**4E-11**
log(Insulin)					0.39	0.52			0.38	0.53
log(Triglycerides)					2.55	**0.04**			2.22	0.08
log(Glucose)					0.19	0.80			0.07	0.92
HDL Cholesterol					-0.01	0.25			-0.01	0.27
Systolic blood pressure					0.01	0.20			0.01	0.20
Diastolic blood pressure					0.01	0.61			0.01	0.59
log(Waist-to-hip ratio)					5.86	0.10			5.67	0.11
Number of MetSyn symptoms							0.42	**8E-5**		

Supplementary Fig. S7. Age adjusted blood cell counts versus phenotypic age acceleration in the Women's Health Initiative (BA23 data). DNAm PhenoAgeAccel (x-axis) is associated with various measures of abundance of blood cell counts. (A) plasma blasts (activated B cells); (B) percentage of exhausted CD8+ T cells (defined as CD8+CD28-CD45RA-); (C) naïve CD8+ T cell count; (D) naïve CD4+ T cell count; E) proportion of CD+8 T cells; F) proportion of CD4+ helper T cells; G) proportion of B cells; H) monocytes and I) proportion of granulocytes (mainly neutrophils). It is not associated with proportion of natural killer cells (results not shown). The correlation coefficient and p-value results from the Pearson correlation test. Points are colored by race/ethnicity (blue=Hispanic, green=African Ancestry, grey=non-Hispanic white). Similar correlations can be found when focusing on individuals of the same ancenstry.

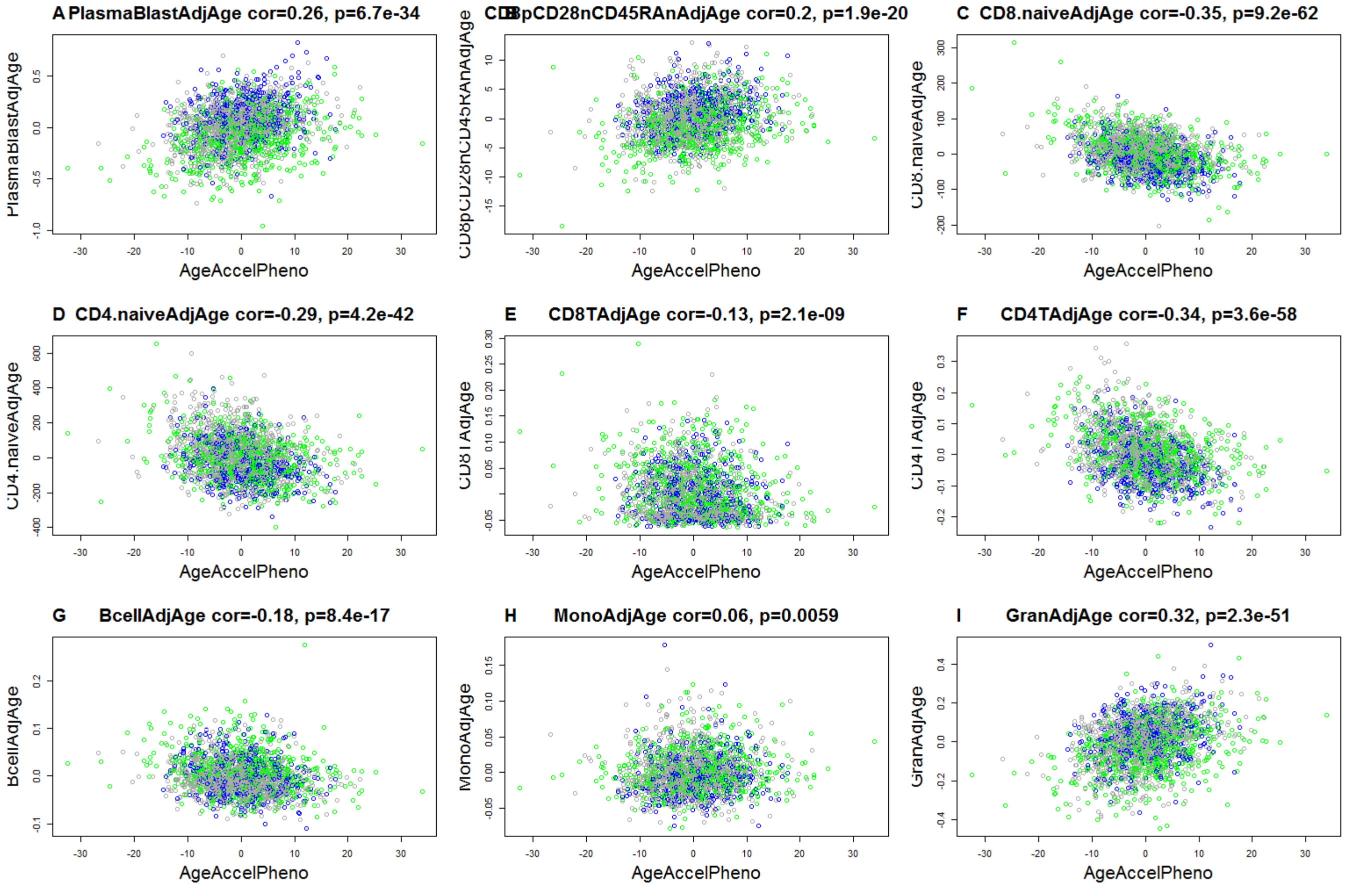

SUPPLEMENTARY FIG. S8. Association between DNAm PhenoAge and LTL.

After adjusting for chronological age, DNAm PhenoAge has a very weak negative correlation with LTL, for the whole population, and stratifying by race/ethnicity and/or sex. This suggests that higher DNAm PhenoAge is modestly related to shorter LTL.

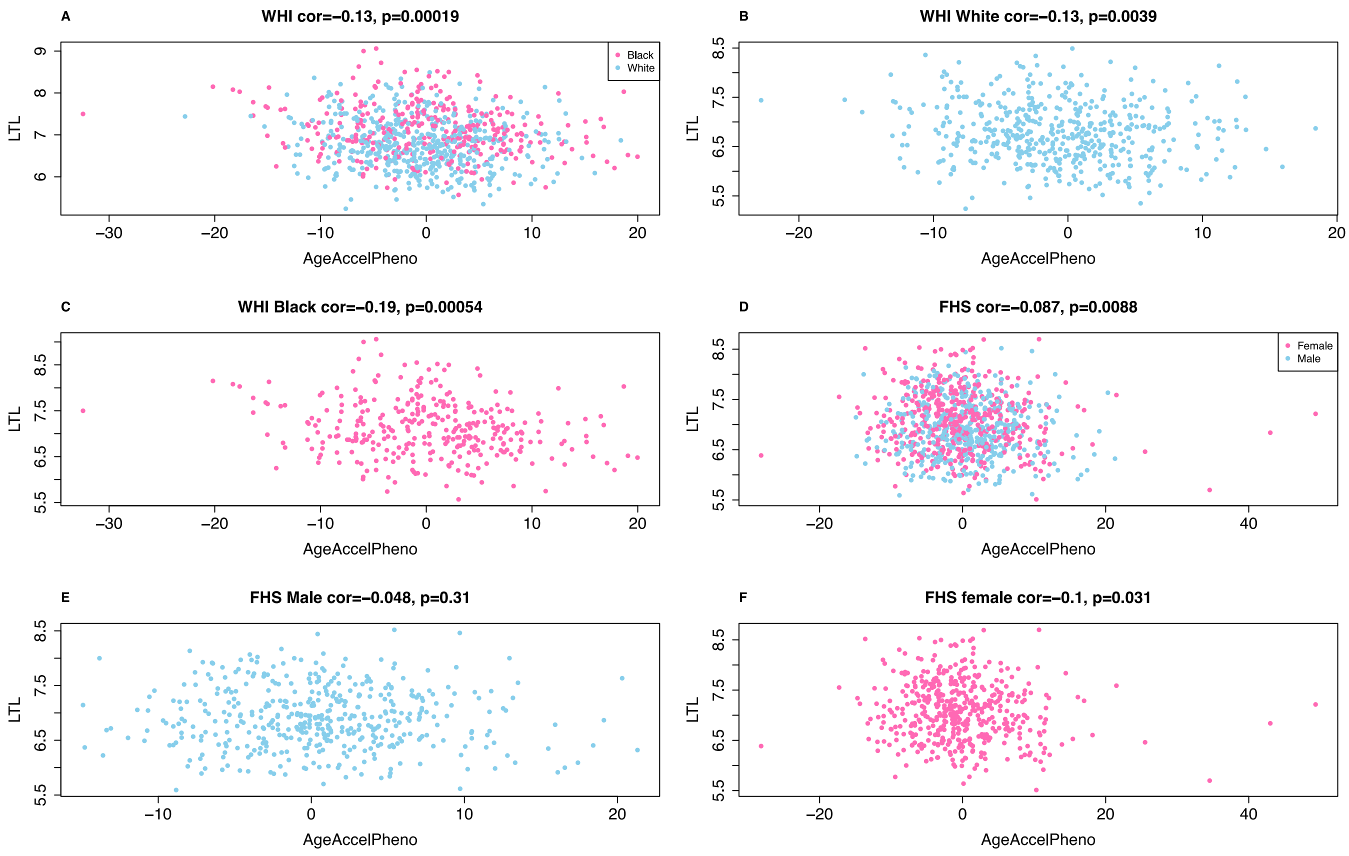

SUPPLEMENTARY FIG. S9. Fixed effects meta analysis of the effect of DNAm phenotypic age acceleration on the hazard of death after adjusting for blood cell counts. DNAm PhenoAge is significantly predictive of all-cause mortality even after accounting for leukocyte proportions. The Cox regression model is adjusted for chronological age, race/ethnicity, smoking pack years, and imputed blood cell counts (exhausted CD8+ T cells, naïve CD8+ T cells, CD4T cells, natural killer cells monocytes, granulocytes). The meta-analysis p value is colored in red. A significant heterogeneity p value (red font) indicates that the hazard ratios differ significantly across studies.

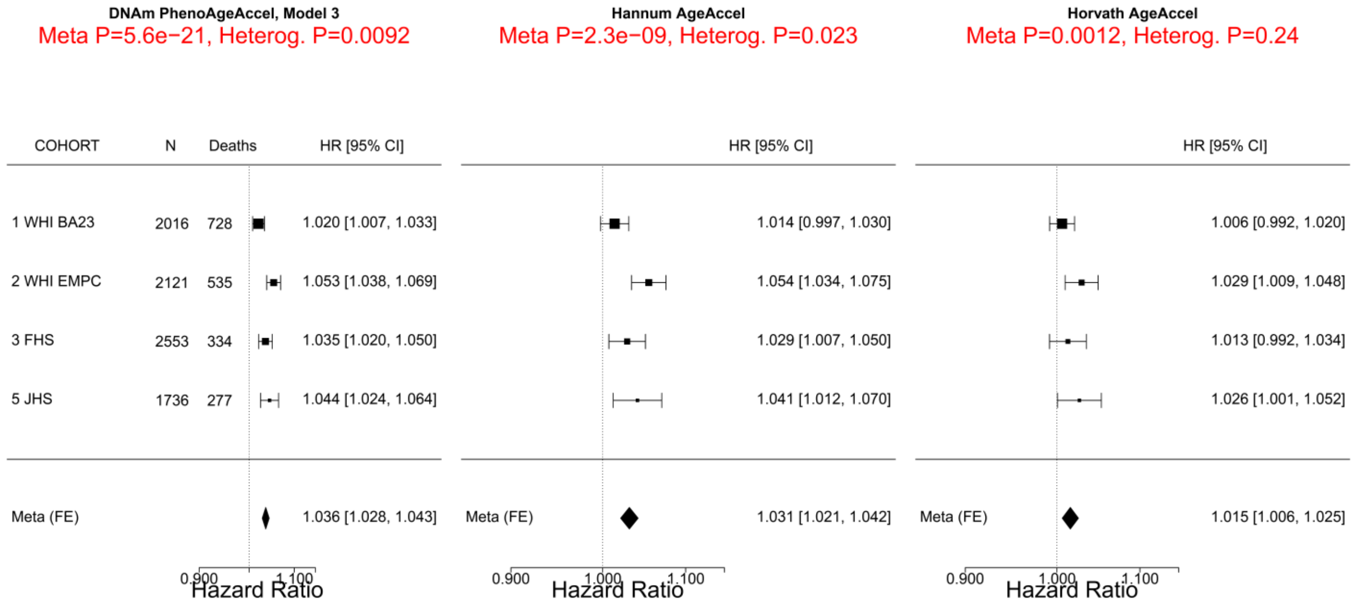

SUPPLEMENTARY FIG. S10. Properties of the 513 CpGs that underly DNAmPhenoAge. In our functional enrichment analysis of the chromosomal locations of the 513 CpGs, we distinguished CpGs with positive age correlation from CpGs with negative age correlation. CpGs with positive age correlation exhibited a lower variance but a similar mean methylation level compared to CpGs with negative age correlation (B,C). The 149 CpGs whose age correlation exceeded 0.2 tended to be located in CpG islands (E) and were significantly enriched with polycomb group protein targets (p=8.7E-5, D). A) Each CpGs was correlated with chronological age in whole blood. The histogram shows the correlation coefficients.

Statistical comments: To avoid biased enrichment results, it is important to use the correct background set of CpGs when it comes to characterizing the properties of the 513 CpGs. The set of CpGs on the Illumina 27K array is the appropriate background set because this set of CpGs was used when training the DNAmPhenoAge estimator. While we used the Illumina 450K array to measure DNA methylation levels, we only used a small subset of the CpGs (namely those located both on the Illumina 27K and the EPIC array) as training set when developing DNAmPhenoAge. We discarded most of the CpGs on the Illumina 450K array when it came to training the DNAmPhenoAge estimator because, surprisingly, the resulting DNAmPhenoAge estimator exhibited more significant predictive associations with lifespan than DNAm estimators built using the full set of CpGs on the Illumina 450K array.

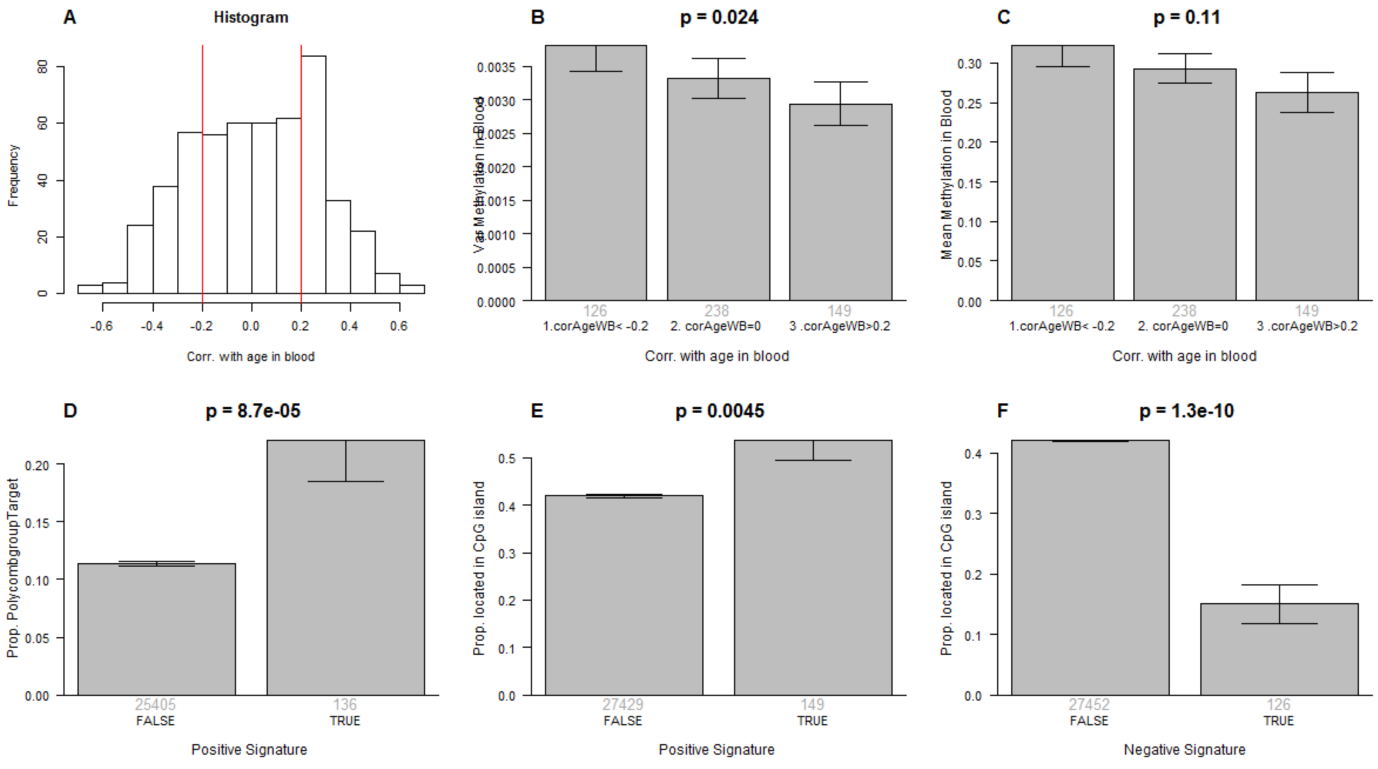

SUPPLEMENTARY FIG. S11: Correlation between 1) gene-DNAm PhenoAgeAccel correlations and 2) gene-chronological age correlations. Genes have similar correlations with chronological age, as they do with DNAm PhenoAgeAccel (DNAm PhenoAge adjusted for age). This suggest that genes that tend to increase as a function of age, tend to be upregulated in persons who are epigenetically older than expected, compared to other of the same age. Conversely, genes that show decreased expression with age, are downregulated among those who are epigenetically older than expected. This can be taken to signify that age-related transcriptional alterations are further exacerbated for those with higher DNAmPhenoAge, relative to their chronological age. However, these cross sectional association studies do not allow us dissect cause and effect relationships between gene transcripts and DNA methylation changes.

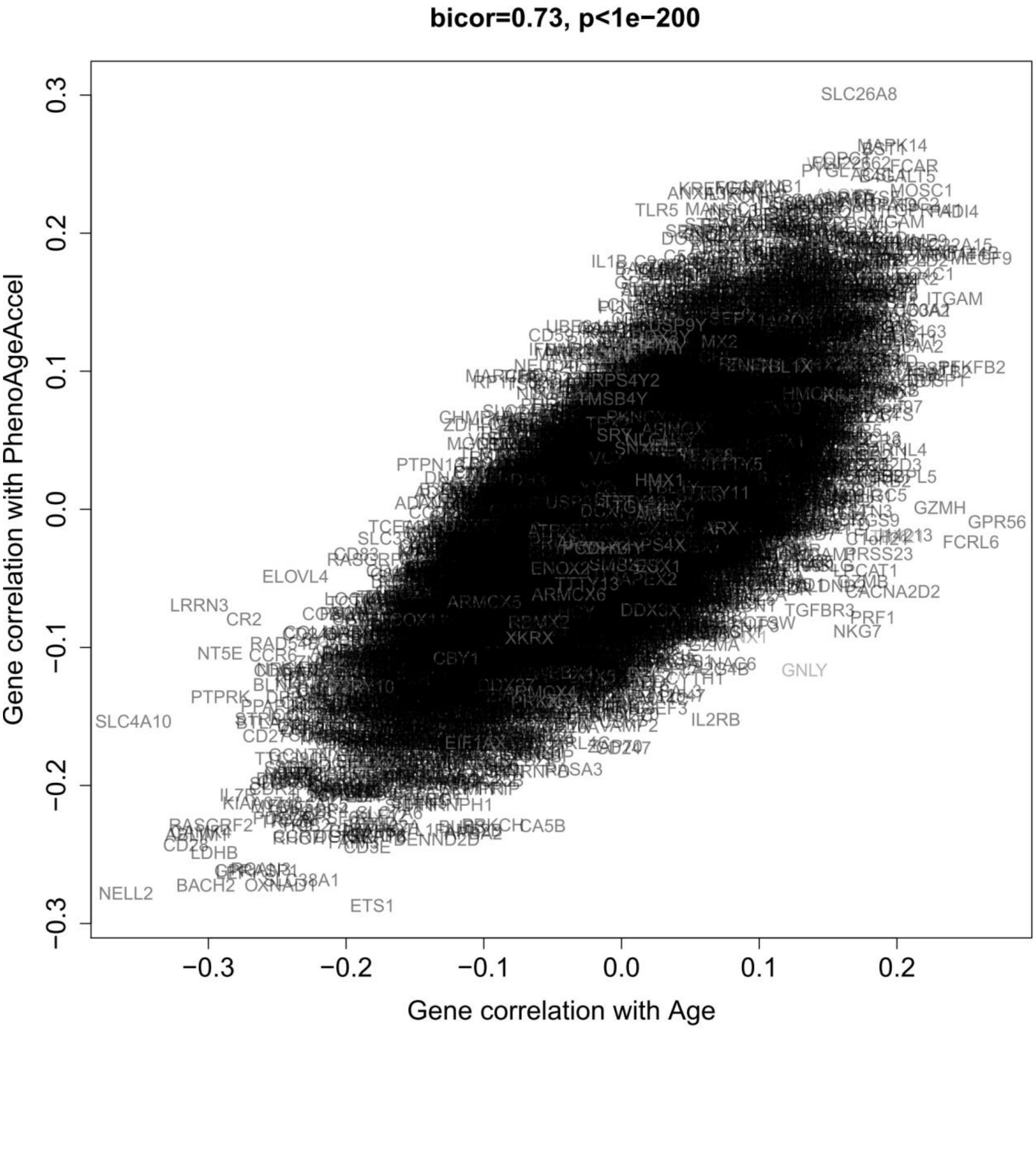

SUPPLEMENTARY FIG. S12: Correlation between elastic net beta coefficients and age correlations. Overall, the coefficients (weights) for the 513 CpGs in the DNAm PhenoAge score (x-axis) are not highly correlated with univariate age correlations (y-axis). Moreover, many of the CpGs shown in blue have little to know age correlation, while having some of the highest weights.

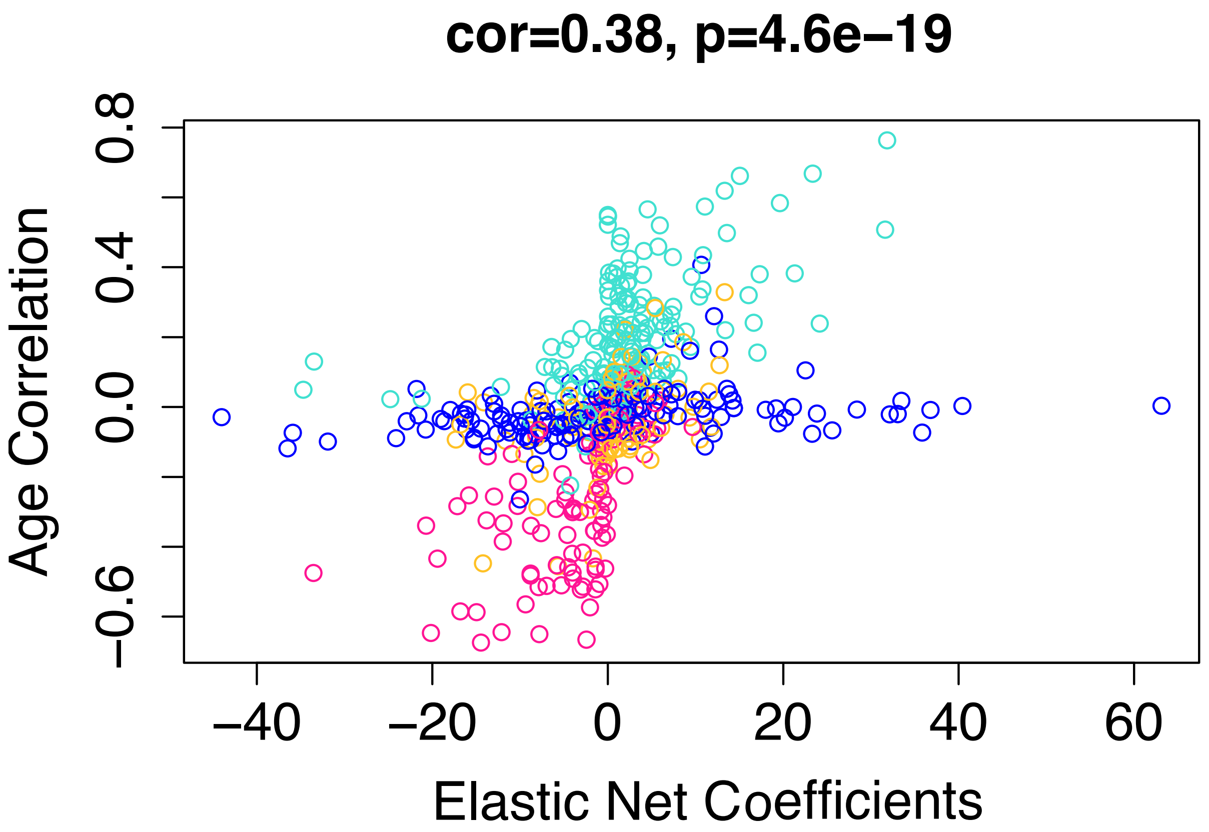

SUPPLEMENTARY FIG. S13. Partial likelihood versus log(lambda) parameter for elastic net proportional hazard model. Ten-fold cross-validation was employed to select the parameter value, lambda, for the penalized regression. In order to develop a sparse phenotypic age estimator (the fewest biomarker variables needed to produce robust results) we selected a lambda of 0.0192, which represented a one standard deviation increase over the lambda with minimum mean-squared error during cross-validation. Of the forty-two biomarkers included in the penalized Cox regression model, this resulted in ten variables (including chronological age) that were selected for the phenotypic age predictor.

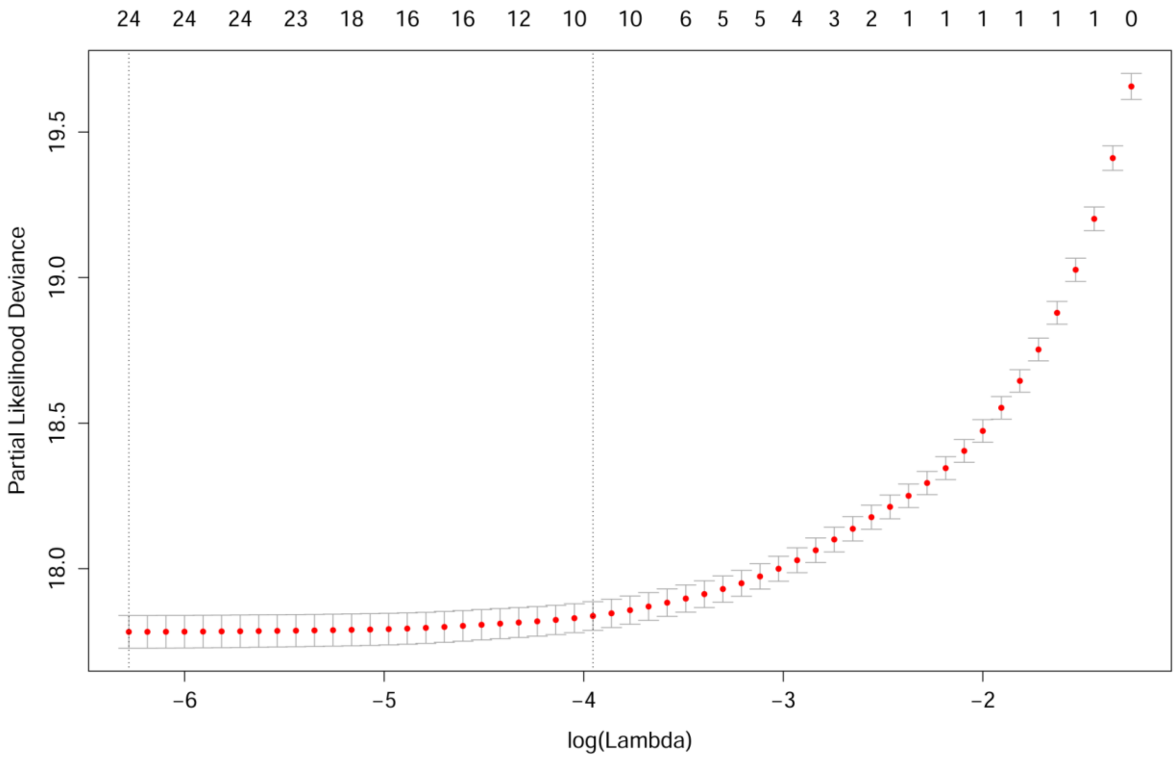

SUPPLEMENTARY FIG. S14. Partial likelihood versus log(lambda) parameter for elastic net regression

The CpGs used in the elastic net represent those that are found on the Illumina Infinium 450k chip, the EPIC chip, and the Illumina Infinium 27k chip. Lambda was selected using 10-fold cross-validation; however, given that sparseness was not a goal with this model, the lambda with the minimum mean-squared error was selected (lambda=0.35). This lambda, produced a model in which phenotypic age is predicted by DNAm levels at 513 CpGs.

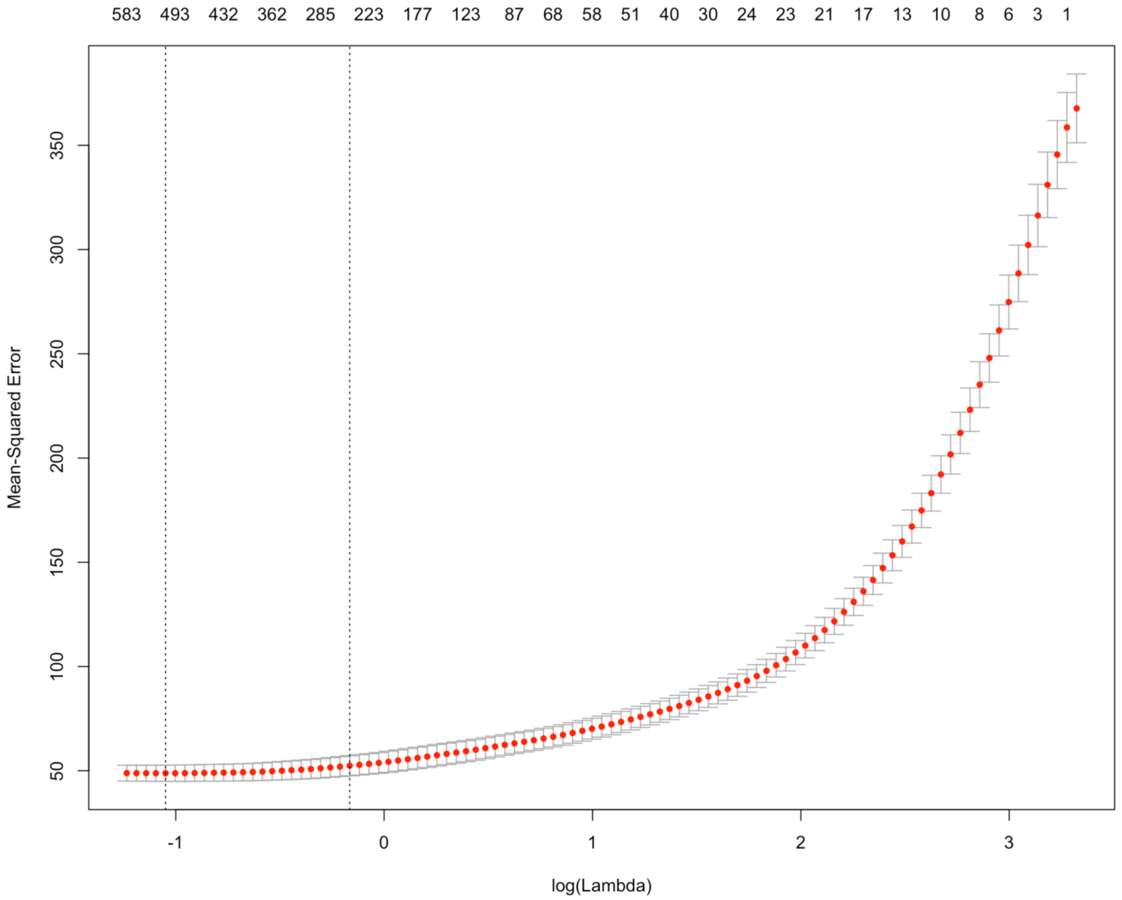

SUPPLEMENTARY FIG. S15: Scatterplots of top genes vs DNAm PhenoAgeAccel and chronological age in the FHS PBMC data. Scatterplots depict the associations (negative in blue and positive in red) between expression in top genes and either DNAm PhenoAgeAccel (top six panels), or chronological age (bottom six panels). Results suggest that genes relate similarly to age adjusted DNAm PhenoAgeAccel and chronological age.

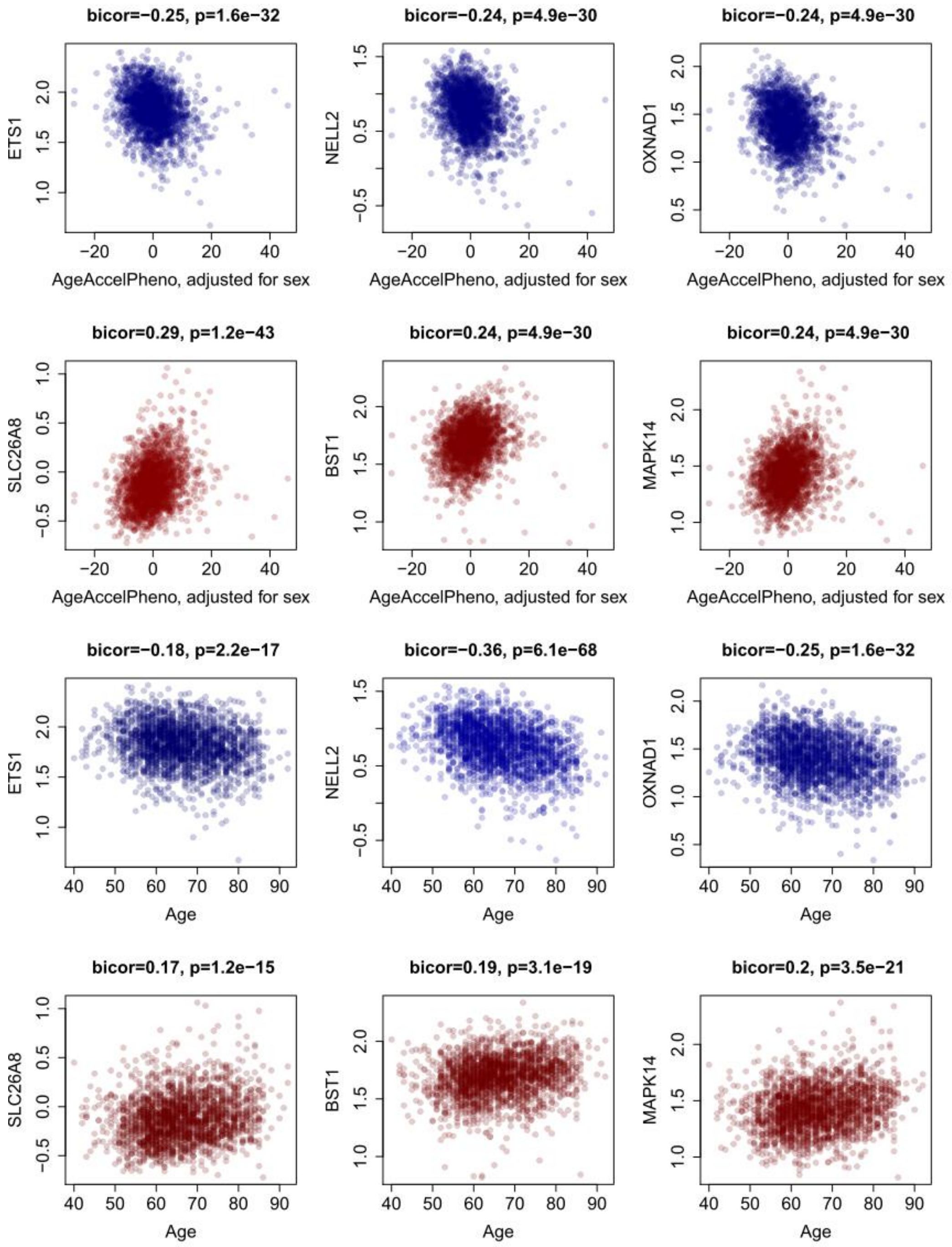

SUPPLEMENTARY FIG. S16: Scatterplots of top genes vs DNAm PhenoAgeAccel and chronological age in the MESA Monocyte data. Scatterplots depict the associations (negative in blue and positive in red) between expression in top genes and either DNAm PhenoAgeAccel (top six panels), or chronological age (bottom six panels). Results suggest that genes relate similarly to age adjusted DNAm PhenoAgeAccel and chronological age.

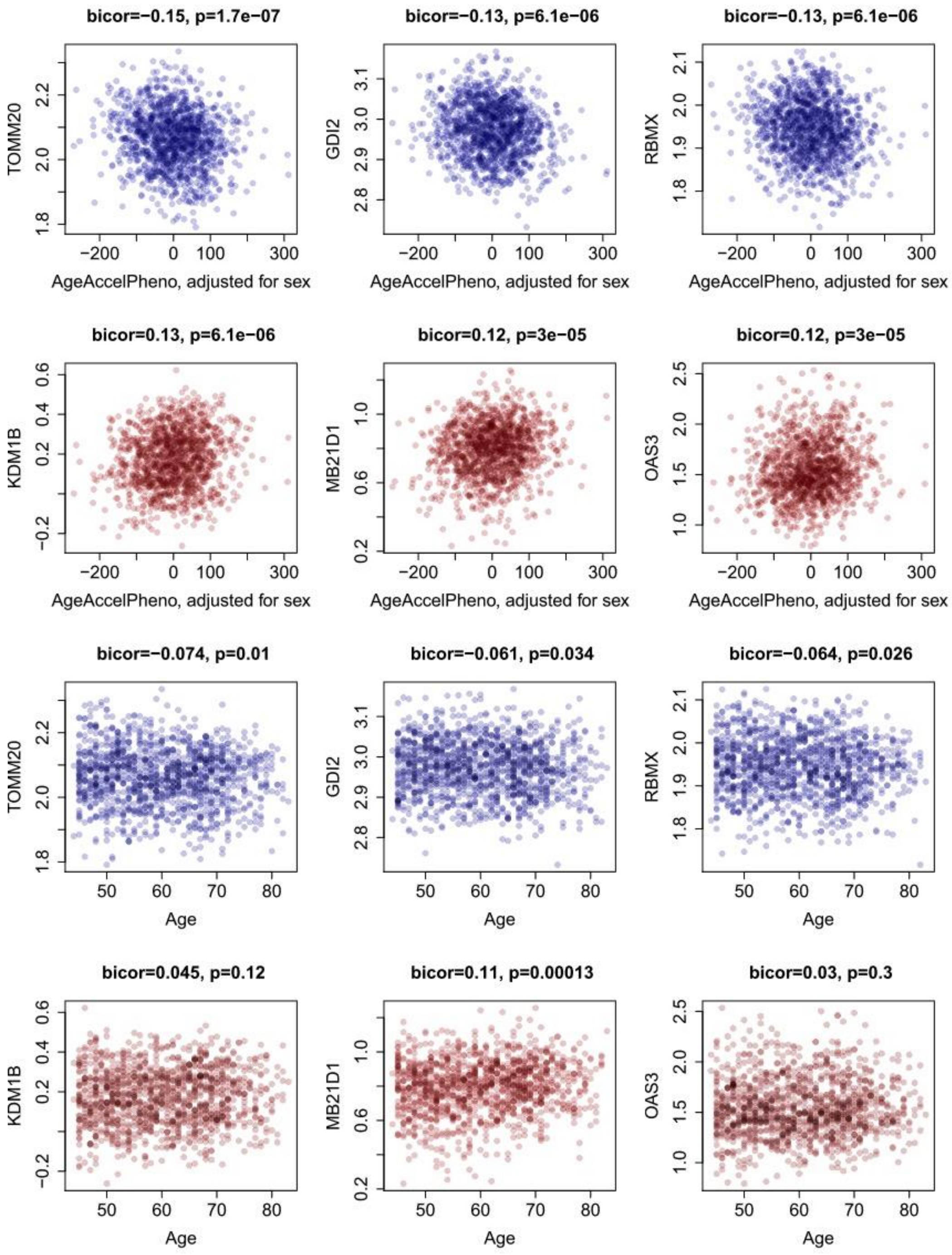

SUPPLEMENTARY FIG. S17. The 513 CpGs from DNAm PhenoAge Mapped to Genomic Locations

CpGs were mapped to genomic locations. Overall, CpGs from the DNAm PhenoAge score do not tend to cluster in specific genomic regions.

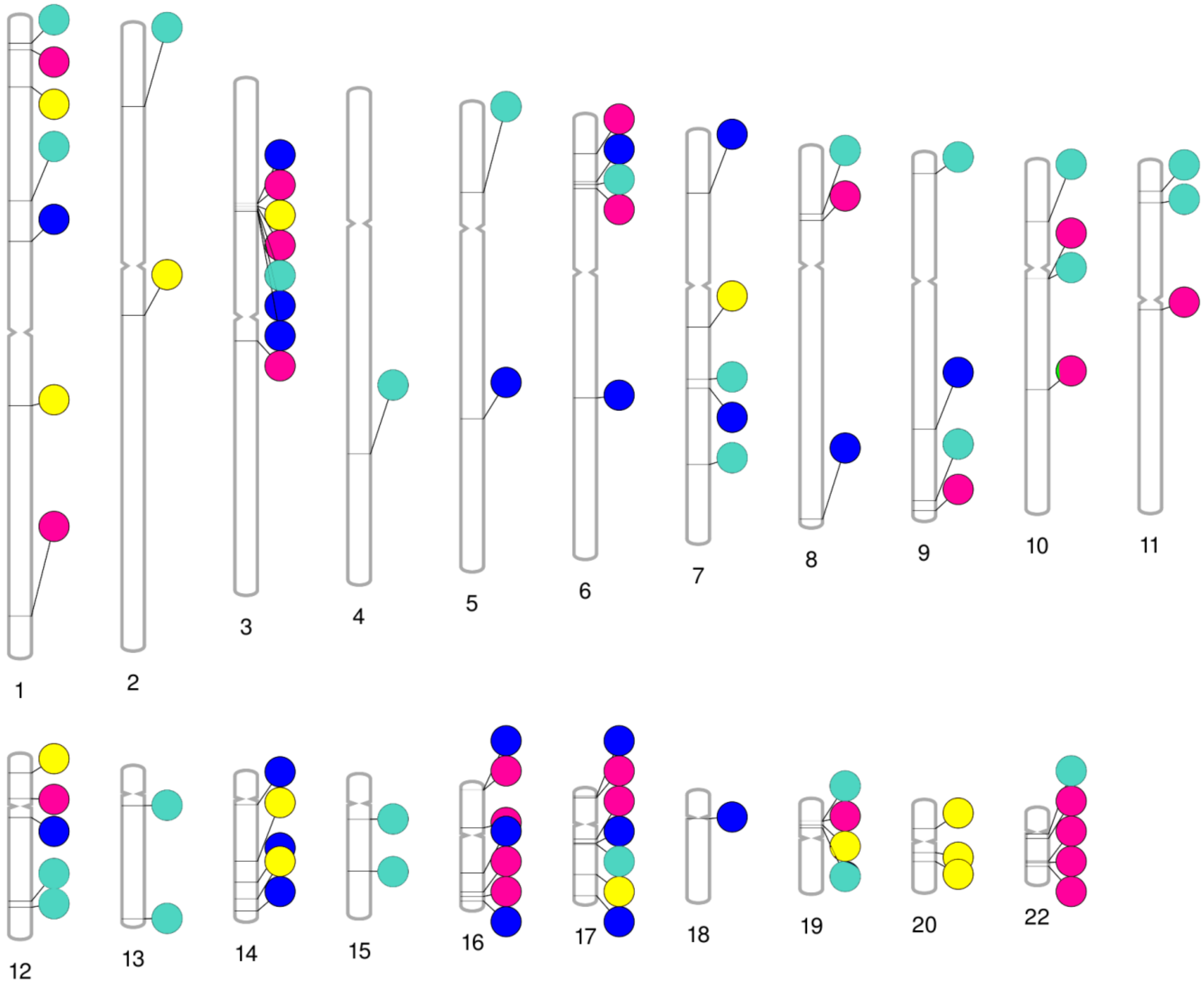

REFERENCES

1. Lee ET, Wang JW: *Statistical Methods for Survival Data Analysis.* Wiley Publishing; 2013.
2. Teschendorff AE, Marabita F, Lechner M, Bartlett T, Tegner J, Gomez-Cabrero D, Beck S: **A beta-mixture quantile normalization method for correcting probe design bias in Illumina Infinium 450 k DNA methylation data.** *Bioinformatics* 2013, **29:**189-196.
3. Horvath S, Gurven M, Levine ME, Trumble BC, Kaplan H: **An epigenetic clock analysis of race/ethnicity, sex, and coronary heart disease.** *Genome Biol* 2016, **17**.
4. Anonymous A: **Design of the Women's Health Initiative clinical trial and observational study. The Women's Health Initiative Study Group.** *Control Clin Trials* 1998, **19:**61-109.
5. Bell B, Rose CL, Damon A: **The Veterans Administration longitudinal study of healthy aging.** *The Gerontologist* 1966, **6:**179-184.
6. Taylor Jr HA, Wilson JG, Jones DW, Sarpong DF, Srinivasan A, Garrison RJ, Nelson C, Wyatt SB: **Toward resolution of cardiovascular health disparities in African Americans: design and methods of the Jackson Heart Study.** *Ethn Dis* 2005, **15:**S6-4.
7. Levine ME, Lu AT, Chen BH, Hernandez DG, Singleton AB, Ferrucci L, Bandinelli S, Salfati E, Manson JE, Quach A, et al: **Menopause accelerates biological aging.** *Proc Natl Acad Sci U S A* 2016, **113:**9327-9332.
8. Ambatipudi S, Horvath S, Perrier F, Cuenin C, Hernandez-Vargas H, Le Calvez-Kelm F, Durand G, Byrnes G, Ferrari P, Bouaoun L, et al: **DNA methylome analysis identifies accelerated epigenetic ageing associated with postmenopausal breast cancer susceptibility.** *Eur J Cancer* 2017, **75:**299-307.
9. Horvath S, Pirazzini C, Bacalini MG, Gentilini D, Blasio AM, Delledonne M, Mari D, Arosio B, Monti D, Passarino G: **Decreased epigenetic age of PBMCs from Italian semi-supercentenarians and their offspring.** *Aging (Albany NY)* 2015, **7**.
10. Li Y, Chen JA, Sears RL, Gao F, Klein ED, Karydas A, Geschwind MD, Rosen HJ, Boxer AL, Guo W, et al: **An epigenetic signature in peripheral blood associated with the haplotype on 17q21.31, a risk factor for neurodegenerative tauopathy.** *PLoS Genet* 2014, **10:**e1004211.
11. Horvath S, Garagnani P, Bacalini M, Pirazzini C, Salvioli S, Gentilini D, DiBlasio A, Giuliani C, Tung S, Vinters H, Franceschi C: **Accelerated Epigenetic Aging in Down Syndrome.** *Aging Cell* 2015, **14**.
12. Kerkel K, Schupf N, Hatta K, Pang D, Salas M, Kratz A, Minden M, Murty V, Zigman WB, Mayeux RP, et al: **Altered DNA Methylation in Leukocytes with Trisomy 21.** *PLoS Genet* 2010, **6:**e1001212.
13. Horvath S, Levine AJ: **HIV-1 infection accelerates age according to the epigenetic clock.** *J Infect Dis* 2015.
14. Horvath S, Ritz BR: **Increased epigenetic age and granulocyte counts in the blood of Parkinson's disease patients.** *Aging (Albany NY)* 2015, **7:**1130-1142.

15. Costello S, Cockburn M, Bronstein J, Zhang X, Ritz B: **Parkinson's Disease and Residential Exposure to Maneb and Paraquat From Agricultural Applications in the Central Valley of California.** *American Journal of Epidemiology* 2009, **169:**919-926.
16. Houseman E, Accomando W, Koestler D, Christensen B, Marsit C, Nelson H, Wiencke J, Kelsey K: **DNA methylation arrays as surrogate measures of cell mixture distribution.** *BMC Bioinformatics* 2012, **13:**86.
17. Horvath S: **DNA methylation age of human tissues and cell types.** *Genome Biol* 2013, **14**.
18. Horvath S, Gurven M, Levine ME, Trumble BC, Kaplan H, Allayee H, Ritz BR, Chen B, Lu AT, Rickabaugh TM, et al: **An epigenetic clock analysis of race/ethnicity, sex, and coronary heart disease.** *Genome Biol* 2016, **17:**171.
19. Almasy L, Blangero J: **Multipoint quantitative-trait linkage analysis in general pedigrees.** *Am J Hum Genet* 1998, **62:**1198-1211.
20. Lee SH, Wray NR, Goddard ME, Visscher PM: **Estimating missing heritability for disease from genome-wide association studies.** *Am J Hum Genet* 2011, **88:**294-305.
21. Visscher PM, Hemani G, Vinkhuyzen AA, Chen GB, Lee SH, Wray NR, Goddard ME, Yang J: **Statistical power to detect genetic (co)variance of complex traits using SNP data in unrelated samples.** *PLoS Genet* 2014, **10:**e1004269.
22. Guintivano J, Aryee MJ, Kaminsky ZA: **A cell epigenotype specific model for the correction of brain cellular heterogeneity bias and its application to age, brain region and major depression.** *Epigenetics* 2013, **8:**290-302.
23. Reynolds LM, Taylor JR, Ding J, Lohman K, Johnson C, Siscovick D, Burke G, Post W, Shea S, Jacobs DR, Jr., et al: **Age-related variations in the methylome associated with gene expression in human monocytes and T cells.** *Nat Commun* 2014, **5:**5366.
24. Horvath S, Erhart W, Brosch M, Ammerpohl O, von Schonfels W, Ahrens M, Heits N, Bell JT, Tsai PC, Spector TD, et al: **Obesity accelerates epigenetic aging of human liver.** *Proc Natl Acad Sci U S A* 2014, **111:**15538-15543.
25. Franceschi C, Bonafè M, Valensin S, Olivieri F, De Luca M, Ottaviani E, De Benedictis G: **Inflamm-aging. An evolutionary perspective on immunosenescence.** *Ann N Y Acad Sci* 2000, **908:**244-254.
26. Florath I, Butterbach K Fau - Muller H, Muller H Fau - Bewerunge-Hudler M, Bewerunge-Hudler M Fau - Brenner H, Brenner H: **Cross-sectional and longitudinal changes in DNA methylation with age: an epigenome-wide analysis revealing over 60 novel age-associated CpG sites.**
27. Johansson A, Enroth S Fau - Gyllensten U, Gyllensten U: **Continuous Aging of the Human DNA Methylome Throughout the Human Lifespan.**
28. Garagnani P, Bacalini Mg Fau - Pirazzini C, Pirazzini C Fau - Gori D, Gori D Fau - Giuliani C, Giuliani C Fau - Mari D, Mari D Fau - Di Blasio AM, Di Blasio Am Fau - Gentilini D, Gentilini D Fau - Vitale G, Vitale G Fau - Collino S, Collino S Fau - Rezzi S, et al: **Methylation of ELOVL2 gene as a new epigenetic marker of age.**
29. Hannum G, Guinney J, Zhao L, Zhang L, Hughes G, Sadda S, Klotzle B, Bibikova M, Fan JB, Gao Y: **Genome-wide methylation profiles reveal quantitative views of human aging rates.** *Mol Cell* 2013, **49**.
30. McLean CY, Bristor D, Hiller M, Clarke SL, Schaar BT, Lowe CB, Wenger AM, Bejerano G: **GREAT improves functional interpretation of cis-regulatory regions.** *Nat Biotechnol* 2010, **28:**495-501.
31. Horvath S: **DNA methylation age of human tissues and cell types.** *Genome Biol* 2013, **14:**R115.

32. Hannum G, Guinney J, Zhao L, Zhang L, Hughes G, Sadda S, Klotzle B, Bibikova M, Fan JB, Gao Y, et al: **Genome-wide methylation profiles reveal quantitative views of human aging rates.** *Mol Cell* 2013, **49:**359-367.
33. Levine M, Lu A, Quach A, Chen B, Assimes T, Bandinelli S, Hou L, Baccarelli A, Stewart J, Li Y, et al: **An epigenetic biomarker of aging for lifespan and healthspan.** 2018.
34. Weidner CI, Lin Q, Koch CM, Eisele L, Beier F, Ziegler P, Bauerschlag DO, Jockel KH, Erbel R, Muhleisen TW, et al: **Aging of blood can be tracked by DNA methylation changes at just three CpG sites.** *Genome Biol* 2014, **15:**R24.
35. Lin Q, Weidner CI, Costa IG, Marioni RE, Ferreira MR, Deary IJ, Wagner W: **DNA methylation levels at individual age-associated CpG sites can be indicative for life expectancy.** *Aging (Albany NY)* 2016, **8:**394-401.
36. Horvath S, Pirazzini C, Bacalini MG, Gentilini D, Di Blasio AM, Delledonne M, Mari D, Arosio B, Monti D, Passarino G, et al: **Decreased epigenetic age of PBMCs from Italian semi-supercentenarians and their offspring.** *Aging (Albany NY)* 2015, **7:**1159-1170.
37. Levine AJ, Quach A, Moore DJ, Achim CL, Soontornniyomkij V, Masliah E, Singer EJ, Gelman B, Nemanim N, Horvath S: **Accelerated epigenetic aging in brain is associated with pre-mortem HIV-associated neurocognitive disorders.** *J Neurovirol* 2016, **22:**366-375.
38. Ahrens M, Ammerpohl O, von Schönfels W, Kolarova J, Bens S, Itzel T, Teufel A, Herrmann A, Brosch M, Hinrichsen H, et al: **DNA Methylation Analysis in Nonalcoholic Fatty Liver Disease Suggests Distinct Disease-Specific and Remodeling Signatures after Bariatric Surgery.** *Cell Metabolism* 2013, **18:**296-302.
39. Horvath S: **Erratum to: DNA methylation age of human tissues and cell types.** *Genome Biol* 2015, **16:**96.
40. Horvath S, Levine AJ: **HIV-1 Infection Accelerates Age According to the Epigenetic Clock.** *J Infect Dis* 2015, **212:**1563-1573.
41. **Webpage:** http://labs.genetics.ucla.edu/horvath/dnamage
